# Micro-RNA Profiling in Human Serum Reveals Compartment-Specific Roles of miR-571 and miR-652 in Liver Cirrhosis

**DOI:** 10.1371/journal.pone.0032999

**Published:** 2012-03-07

**Authors:** Christoph Roderburg, Tobias Mollnow, Brenda Bongaerts, Natalia Elfimova, David Vargas Cardenas, Katharina Berger, Henning Zimmermann, Alexander Koch, Mihael Vucur, Mark Luedde, Claus Hellerbrand, Margarete Odenthal, Christian Trautwein, Frank Tacke, Tom Luedde

**Affiliations:** 1 Department of Medicine III, University Hospital RWTH Aachen, Aachen, Germany; 2 Department of Pathology, Maastricht University Medical Center, Maastricht, The Netherlands; 3 Institute for Pathology, University Hospital Cologne, Cologne, Germany; 4 Department of Cardiology and Angiology, University of Kiel, Kiel, Germany; 5 Department of Internal Medicine I, University of Regensburg, Regensburg, Germany; Sudbury Regional Hospital, Canada

## Abstract

**Background and Aims:**

Micro-RNAs (miRNAs) have recently emerged as crucial modulators of molecular processes involved in chronic liver diseases. The few miRNAs with previously proposed roles in liver cirrhosis were identified in screening approaches on liver parenchyma, mostly in rodent models. Therefore, in the present study we performed a systematic screening approach in order to identify miRNAs with altered levels in the serum of patients with chronic liver disease and liver cirrhosis.

**Methods:**

We performed a systematic, array-based miRNA expression analysis on serum samples from patients with liver cirrhosis. In functional experiments we evaluated the relationship between alterations of miRNA serum levels and their role in distinct cellular compartments involved in hepatic cirrhosis.

**Results:**

The array analysis and the subsequent confirmation by qPCR in a larger patient cohort identified significant alterations in serum levels of miR-513-3p, miR-571 and miR-652, three previously uncharacterized miRNAs, in patients with alcoholic or hepatitis C induced liver cirrhosis. Of these, miR-571 serum levels closely correlated with disease stages, thus revealing potential as a novel biomarker for hepatic cirrhosis. Further analysis revealed that up-regulation of miR-571 in serum reflected a concordant regulation in cirrhotic liver tissue. In isolated primary human liver cells, miR-571 was up-regulated in human hepatocytes and hepatic stellate cells in response to the pro-fibrogenic cytokine TGF-β. In contrast, alterations in serum levels of miR-652 were stage-independent, reflecting a concordant down-regulation of this miRNA in circulating monocytes of patients with liver cirrhosis, which was inducible by proinflammatory stimuli like bacterial lipopolysaccharide.

**Conclusion:**

Alterations of miR571 and miR-652 serum levels in patients with chronic liver disease reflect their putative roles in the mediation of fibrogenic and inflammatory processes in distinct cellular compartments involved in the pathogenesis of liver cirrhosis.

## Introduction

Many chronic liver diseases are still not sufficiently treatable and often progress to liver cirrhosis representing the major risk factor for the development of hepatocellular carcinoma (HCC) [1]. Despite recent advances e.g. in the treatment of viral hepatitis, pharmacological strategies to prevent fibrogenesis, to facilitate the reversal of fibrosis/cirrhosis or to prevent the complications of advanced hepatic cirrhosis are still limited [2], underlining the need to establish a better understanding of the molecular mechanisms underlying the pathogenesis of hepatic cirrhosis.

Micro-RNAs (miRNAs) are small, non-coding, 21–23 nucleotide long RNAs that negatively regulate gene expression by base pairing with the 3′-untranslated region (UTR) of their target mRNAs [3]. miRNAs are involved in highly regulated processes such as cell injury, proliferation or carcinogenesis [4], [5]. In the liver, previous studies have shown that miRNAs play a fundamental role in acute liver injury, viral hepatitis or hepatocarcinogenesis [6], [7], [8], [9]. In addition, systematic array approaches on liver tissue from mice and primary hepatic stellate cells (HSCs) recently revealed important functional roles of certain miRNAs in hepatic fibrogenesis. As such, it was shown that members of the miR-29 family integrate pro-fibrogenic and pro-inflammatory signals in hepatic stellate cells and control expression of various extracellular matrix genes during hepatic fibrogenesis [10], [11], [12]. Since miRNAs control networks of target genes and are thus promising therapeutic targets, such findings are of high potential for the establishment of miRNA-based antifibrotic therapies, which have already been tested in other organs like the heart [13].

Previous studies on the role of miRNAs in chronic liver disease have focused on certain miRNAs identified in screening approaches on rodent liver samples or hepatic stellate cell cultures [10], [11], [12], [14]. However, some members of the broad spectrum of human miRNAs are not yet established in rodent array systems. Moreover, chronic liver disease and liver cirrhosis represent systemic diseases with regulatory processes not only occurring in the liver but also in other cellular compartments like immune cells [15]. Therefore, alternative experimental approaches are needed, which employ primary human specimen and acknowledge chronic liver disease as a systemic disorder influenced by distinct cellular compartments.

It has recently become evident that certain disease conditions are associated with alterations in serum levels of miRNAs [16]. Despite the potential of these findings for the establishment of novel biomarkers, serum-level alterations of miRNAs might reflect important regulatory processes occurring in distinct cellular compartments involved in disease pathogenesis. In line with this hypothesis, we recently demonstrated that the functional role of miR-29 in liver fibrosis correlated with a significant decrease of miR-29 serum levels [10]. Therefore, in the present study we performed a comprehensive, unbiased array approach on serum samples from patients and healthy controls in order to identify novel miRNAs that play a role in chronic liver disease and liver cirrhosis.

## Materials and Methods

### Patients and controls

The study protocol was approved by the local ethics committee (ethics committee of University Hospital Aachen, RWTH Aachen), and written informed consent was obtained from each patient. The study was conducted according to the principles expressed in the Declaration of Helsinki.

We studied 67 patients with chronic liver diseases [15]. Characteristics of the study cohort are summarized in table 1. Importantly, patients were excluded in case of systemic inflammatory response syndrome (SIRS) or sepsis, HIV-infection, systemic steroid medication (prednisolone >7.5 mg/d or equivalent doses) and malignant tumor(s) except for hepatocellular carcinoma. As a control group, 17 matched healthy volunteers were recruited from the local blood transfusion institute that had normal aminotransferase activities, no history of liver disease or alcohol abuse and were tested negative for HBV, HCV and HIV infections.

**Table 1:** Clinical data of patients with liver cirrhosis.

				Child-Pugh stages of liver cirrhosis		
	Controls	All patients	no cirrhosis	Child A	Child B	Child C
**[n(%)]**	17	67	6 (9%)	25 (37.3%)	10 (14.9%)	26 (38.8%)
**Gender (m/f) [n(%)]**	12/5	43/24	2/4	16/9	6/4	19/7
**Age (years)**	46 (23–68)	58 (29–82)	39 (29–56)	62 (37–82)	57 (40–77)	55 (38–81)
**Etiology [n(%)]**						
**Viral Hepatitis**		18 (26.9%)	2 (33.3%)	9 (36%)	5 (50%)	2 (7.7%)
**Biliary**		5 (7.5%)	0 (0%)	2 (8%)	2 (20%)	1 (3.8%)
**Alcohol**		43 (64.2%)	4 (66.7%)	13 (52%)	3 (30%)	23 (88.5%)
**Cryptogenic**		1 (1.5%)	0 (0%)	1 (4%)	0 (0%)	0 (0%)
**Complications [n(%)]**						
**Ascites**		28 (41.8%)	1 (1.6%)	4 (16%)	6 (60%)	17 (65.4%)
**Esophageal varices**		35 (52.2%)	0 (0%)	10 (40%)	6 (60%)	19 (73.1%)
**Encephalopathy**		10 (14.9%)	0 (0%)	0 (0%)	1 (10%)	9 (34.6%)

All blood samples were taken by one physician, and strictly the same protocol was applied to all samples (centrifugation time, materials, etc.). Furthermore a “needle to freezer time” of less than 30 min was defined for all samples. All samples were handled at 4°C until storing them for final analysis at −80°C. RNA extraction and cDNA transcription was performed simultaneously for all samples.

For tissue-specific miRNA-regulation analysis, samples were taken from explanted cirrhotic livers (n = 13) from patients that underwent liver transplantation with different disease etiologies (Data S1) [15]. As controls, liver tissues were obtained from patients undergoing partial liver resection for hepatic metastases of colorectal cancer. Only liver tissues judged as non-cancerous by local pathologists and without histological evidence for both liver fibrosis and inflammation were used to serve as control tissue [15].

### miRNA isolation from serum

Total RNA, including miRNA, was extracted from serum samples using Phenol-Chloroform extraction as previously described [10], [11], [12]. In short, 400 µl serum was spiked with 20 nM miScript miRNA mimic SV40 (Qiagen) for sample normalization according to references [6], [17], [18], [19], [20] (Figure S1). 800 µl Phenol (Qiazol, Qiagen, Hilden; Germany) and 200 µl Chloroform were added to the sample and mixed vigorously for 15 sec followed by incubation at room temperature for 10 min. Samples were centrifuged for 15 min at 12,000 g until complete phase separation. The aqueous phase, containing total RNA, was precipitated with 500 µl 100% Isopropanol and 20 µg Glykogen (Fermentas, St. Leonroth; Germany) over night at −20°C. After centrifugation at 4°C for 15 min (12,000 g) the pellets were washed once with 70% ethanol. Precipitated RNA was resuspended in 30 µl RNase free water (Ambion, Austin, TX). To assess the quantity and quality of RNA, the samples were measured using a NanoDrop spectrophotometer (NanoDrop, Wilmington, DE) and by microfluidic electrophoresis using a Small RNA Chip for Agilent's Bioanalyzer (Agilent Technologies, Waldbronn).

### Microarray

Total RNA was extracted as described above. For cDNA synthesis, 1,5 ng of total RNA was transcribed in a 10-µL volume with the TaqMan microRNA RT kit (Applied Biosystems, Foster City, CA) according to the manufacturer's recommendations. Because of generally low RNA concentrations in serum, an additional pre-amplification step for miRNAs was performed using the MegaPlex RT & PreAmp Human Pool Set (Applied Biosystems). 2,5 µl cDNA was used for the pre-amplification reaction and the pre-amplification product was diluted for further analysis according to the manufacturer's recommendations. For the miRNA screening, quantitative real-time PCR was performed with the TaqMan human miRNA array pool A and B v2.0 (Applied Biosystems). 9 µL of the diluted pre-amplification product was used in each of the real-time PCR arrays. P-value adjustment was performed using the Bejamini-Hochberg method. All analysis have been performed MIAME-compliant and raw data were deposited in a MIAME- compliant database (ArrayExpress), as detailed on the MGED Society website http://www.mged.org/Workgroups/MIAME/miame.html. The accession number will be received during the review.

### Isolation of HSC and hepatocytes from human livers

Primary human HSC were isolated from tissue samples obtained from patients undergoing partial liver resection. Experimental procedures were performed according to guidelines of the local ethics committee with patient's informed consent. Primary HSC were isolated using EGTA/collagenase perfusion and pronase incubation, and seperation from nonparenchymal liver cells was attained by arabinogalactan gradient ultracentrifugation as described previously [17]. HSC were cultured in Dulbecco's modified Eagle's medium supplemented with 10% fetal calf serum and 100 U/ml penicillin/streptomycin. Stimulation of the HSC was carried out on 6-well-plates with 10 ng/mL recombinant human TGF-β1 (R&D Systems) and as control, cells were left untreated.

Primary human hepatocytes were isolated and cultivated in serum-free medium (DMEM supplemented with 4.5 g/l glucose, 0.4 ng/ml hydrocortisone, 0.415 mU/ml insulin, 2 mM glutamine, and 100 U/ml penicillin/streptomycin) as previously described [21].

### Isolation of monocytes and lymphocytes from human blood

Blood of healthy donors was collected in EDTA tubes and directly applied to PBMC isolation by Ficoll Density Gradient (LSM-1077, PAA) and further CD14^+^CD16^−^ and CD14^+^CD16^+^ monocytes were selectively purified by MACS methodology using ‘Monocyte-isolation-Kit-II’ and ‘CD16^+^-monocytes-isolation-kit’, respectively (Miltenyi). Lymphocytes serving as control cells were isolated from PBMC after depletion of monocytes with anti-CD14 microbeads.

**For further materials and methods please see Data S1.**

## Results

### Systematic profiling of serum miRNA levels in patients with distinct entities of liver cirrhosis

**Figure 1:**
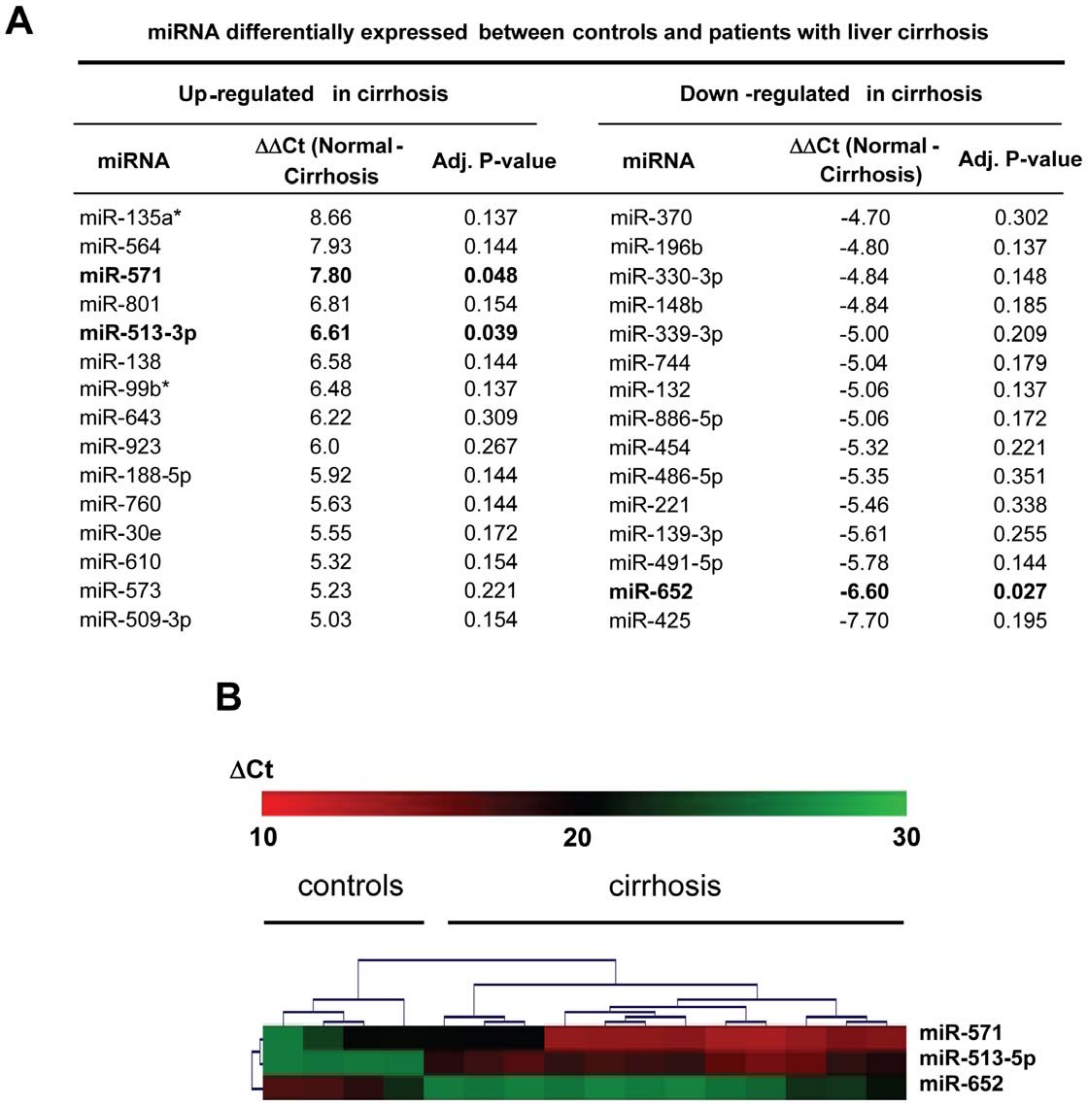
Systematic profiling of serum miRNA levels in patients with distinct entities of liver cirrhosis. (A) Microarray analysis for miRNA levels was performed using RNA extracts from serum of four healthy subjects as control and patients with liver cirrhosis of different etiologies and stages of disease (for detailed clinical parameters see Data S1). The fifteen miRNAs with strongest up- or down-regulation are depicted. Significantly regulated miRNAs are highlighted in bold type. (B) Hierarchical cluster analysis of the significantly regulated miRNAs miR-513-3p; miR-571; miR-652; bright green: under-expression; black: no change; bright red: over-expression.

We performed a systematic array analysis on miRNA-serum levels of patients with liver cirrhosis from different entities and compared them to expression profiles of healthy controls (Figure 1 A). The screening cohort consisted of four age- and sex-matched patients with histologically and clinically confirmed compensated alcoholic liver cirrhosis (Child-Pugh class A), four patients with decompensated alcoholic cirrhosis (Child-Pugh class C), four patients with compensated hepatitis C-related cirrhosis (Child-Pugh class A) and four healthy volunteers as controls (Data S1). While several miRNAs with high-fold-changes were detected, only three of the individual miRNAs represented on the microarray showed significantly diverging levels between healthy controls and liver cirrhosis patients. Of these three miRNAs, levels of miR-513-3p and miR-571 were significantly increased while levels of miR-652 were significantly decreased (Figure 1 A). Array results for these latter three as well as three other representative miRNAs (miR-10a, mir-29a and miR-221) were confirmed by qPCR (Figure S2 A, B). Furthermore, a hierarchical cluster analysis for miR-513-3p, miR-571 and miR-652 revealed two distinct subsets, one containing the control samples and the other containing all cirrhosis patients included into the array analysis (Figure 1 B), thus confirming the different abundance between patients and controls.

Apart from these three significantly regulated miRNAs, we identified a panel of miRNAs with divergent pattern between patients and controls in our array analysis, of which a striking number had been previously studied in the context of liver disease and organ fibrosis, including miR-99 (up) [22], miR-30e (down) [23], miR-132 (up) [24], miR-196b (down) [25] and miR-221 (up) [26] (Figure 1 A). However, only miR-513-3p, miR-571 and miR-652 reached statistical significance (Figure 1 B). Thus, by applying a systematic array approach, we identified a subset of miRNAs differentially regulated in sera of patients with liver cirrhosis of hepatitis C and alcoholic etiology.

**Figure 2:**
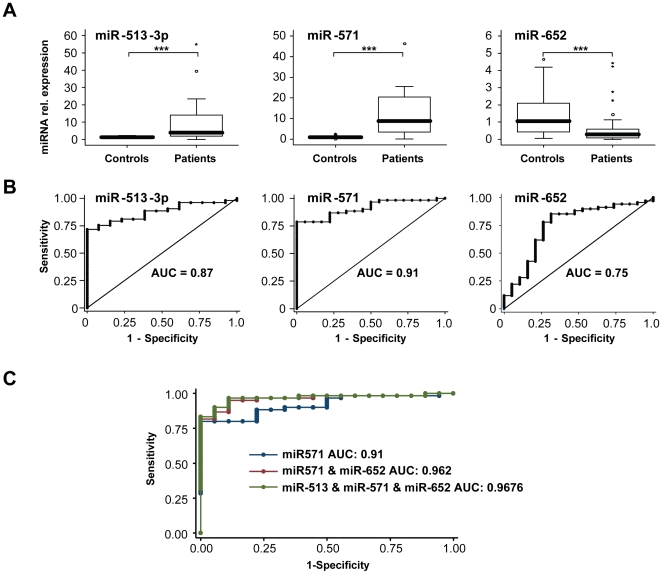
Serum levels of miR-513-3p, miR-571 and miR-652 are significantly altered in the serum of patients with chronic liver disease and liver cirrhosis. (A) Serum levels of the three significantly regulated miRNAs (miR-513-3p; miR-571; miR-652) were analyzed by qPCR in a collective of 17 healthy controls and 67 patients with chronic liver disease and liver cirrhosis. Results are depicted as box plot. The thick line represents the median of relative expression. (B) Receiver operating characteristic (ROC) curve analysis displaying the diagnostic power in predicting cirrhosis of these miRNAs when analyzed as single markers (area under the curve (AUC) 0.87; 0.91; 0.75). (C) ROC curve analysis displaying the diagnostic power in predicting cirrhosis of different combinations of these miRNAs (AUC 0.91; 0.962; 0.9676). **P<0.001.

Next, we analyzed serum levels of miR-513-3p, miR-571 and miR-652 in a cohort of 17 healthy controls and 67 patients with chronic liver diseases by qPCR analysis (Table 1). In this larger collective, we observed a significant alteration for miR-513-3p, miR-571 and miR-652 in patients with liver cirrhosis, which was concordant to the previous findings in the array analysis (Figure 2 A). In addition, three other miRNAs (miR-10a, miR-29a, miR-221), which were strongly but not significantly altered or unaltered in the array, showed a concordant and significant regulation in the larger collective (Figure S2 C). Of note these results could be confirmed in a second, independent collective of patients with liver cirrhosis that were compared with a collective of patients treated for non-hepatic diseases (Figure S3). Moreover, we validated our results by analyzing serum levels of certain miRNAs that were previously shown to be altered in cirrhotics (miR-21, miR-34a, miR-122) [17], [27], [28]. In line with these previous findings, qPCR analysis revealed an up-regulation of miR-34a and miR-122 in our larger qPCR-cohort, while levels of miR-21 were unchanged (Figure S4).

Finally, ROC curve analysis showed that miR-513-3p, miR-571 and miR-652 were highly predictive for the presence of liver disease/cirrhosis (Figure 2 B). Their diagnostic accuracy could even be increased, when miRNA serum levels were combined (Figure 2 C) and were dramatically superior to that of classical markers such as platelets, INR or albumin (AUROC about 0.6), when used in the same setting. Thus, the confirmation of our array results in a large cohort of liver disease patients by qPCR indicated an involvement of miR-513-3p, miR-571 and miR-652 in the pathogenesis of liver cirrhosis.

### Association of miRNA-serum-patterns with etiology and stage of cirrhosis

**Figure 3:**
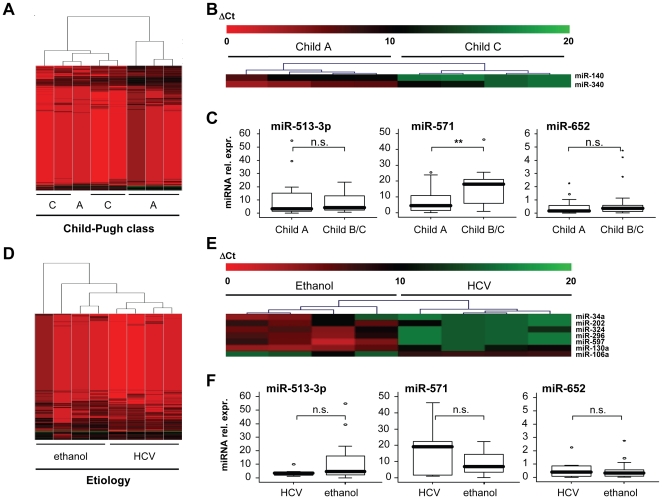
Correlation of miRNA serum levels with Child-Pugh class and etiology of liver disease. (A) Microarray data of the serum of eight patients with alcoholic liver cirrhosis at different stages of disease (Child-Pugh A and Child-Pugh C) in a hierarchical cluster analysis; bright green: under-expression; black: no change; bright red: over-expression. (B) Hierarchical cluster analysis of the significantly regulated miRNAs; bright green: under-expression; black: no change; bright red: over-expression. (C) Serum levels of miR-513-3p, miR-571 and miR-652 in the patients with alcoholic liver cirrhosis were analyzed by qPCR. Results are depicted as box plot. The thick line represents median of relative expression. (D) Microarray data of serum of the serum of four patients with ethanol-toxic liver cirrhosis (Child-Pugh A) and the four hepatitis C related cirrhosis (Child-Pugh A) in a hierarchical-cluster-analysis; bright green: under-expression; black: no change; bright red: over-expression. (E) Hierarchical cluster analysis of the significantly regulated miRNAs; bright green: under-expression; black: no change; bright red: over-expression. (F) Serum levels of miR-513-3p, miR-571 and miR-652 in the patients with alcoholic liver cirrhosis (Child-Pugh A) and hepatitis C related cirrhosis (Child-Pugh A) were analyzed by qPCR. Results are depicted as box plot. The thick line represents median of relative expression. **P<0.01.

We next analyzed our array data with respect to miRNA regulations in serum between either different stages of liver cirrhosis or different etiologies in order to identify more specific stage- or etiology-dependent miRNAs, with a focus on the regulation of miR-652, miR-571 and miR-513-3p. By using hierarchical cluster analysis of all miRNAs comparing Child A and C cirrhosis due to alcoholic disease, the samples split into two defined clusters, differentiating patients with compensated from patients with decompensated cirrhosis (Figure 3 A). However, when we tried to define specific single miRNAs distinguishing early and late stages of cirrhosis, only miR-140 and miR-340 were significantly regulated on the array level between both groups (Figure 3 B). The subsequent qPCR analysis from the total cohort of patients confirmed a trend for a differential regulation of miR-140 and miR-340, but did not reach statistical significance (Figure S5). On the other hand, among the previously identified miR-652, miR-571 and miR-513-3p, only serum levels of miR-571 were significantly elevated in patients with Child C compared to Child A cirrhosis (Figure 3 C). Interestingly, serum levels of miR-571 were significantly correlated with biomarkers of liver synthesis capacity such as pseudocholinesterase (PCHE), prothrombin time, international normalized ratio (INR) or coagulation factor V activity (Data S1), raising the possibility that this distinct miRNAs could serve as a novel serum biomarker reflecting disease severity in liver cirrhosis.

To evaluate if serum miRNA patterns are also dysregulated in an etiology-specific way we next compared serum miRNA expression patterns between patients with alcoholic and hepatitis C cirrhosis at the same stage of liver disease (Child-Pugh class A). Again, hierarchical cluster analysis on the array-data revealed distinct clustering between alcoholic and hepatitis C cirrhosis (Figure 3 D). Seven miRNAs in the array analysis collective were regulated significantly different between these two groups (Figure 3 E), but significance was lost when the larger cohort was analyzed by qPCR. Importantly, miR-652, miR-571 and miR-513-3p did not differ dependent on disease etiology (Figure 3 F). Furthermore, to exclude a bias in regulation of these miRNA by co-morbidities or demographic differences, we correlated miR-652, miR-571 and miR-513-3p levels with age, gender, body mass index and serum creatinine concentration. None of these single parameters correlated with these miRNAs (Data S1). Therefore, in line with our initial hypothesis, the gene array approach indeed allowed us to identify three etiology-independent miRNAs specifically regulated in serum of liver disease patients, with miR-571 being a previously unrecognized miRNA that is also closely associated with the stage of liver cirrhosis.

### Serum levels of miR-571 and miR-652 reflect their specific regulation in distinct cellular compartments involved in the pathogenesis of liver cirrhosis

It is presently unclear and controversially debated which molecular mechanisms are responsible for alterations in serum miRNA levels in certain disease conditions. Especially, it is not known if changes in serum levels reflect a passive release of miRNAs during cell death or if they are actively secreted and might play a functional role as “messengers” [29], [30]. We thus aimed at assessing if divergent patterns of the identified set of serum miRNAs correlated with their regulation in the cellular compartments that are most likely involved in the initiation and promotion of liver cirrhosis, namely liver tissue as well as circulating immune cells such as monocytes or lymphocytes [15], [31].

**Figure 4:**
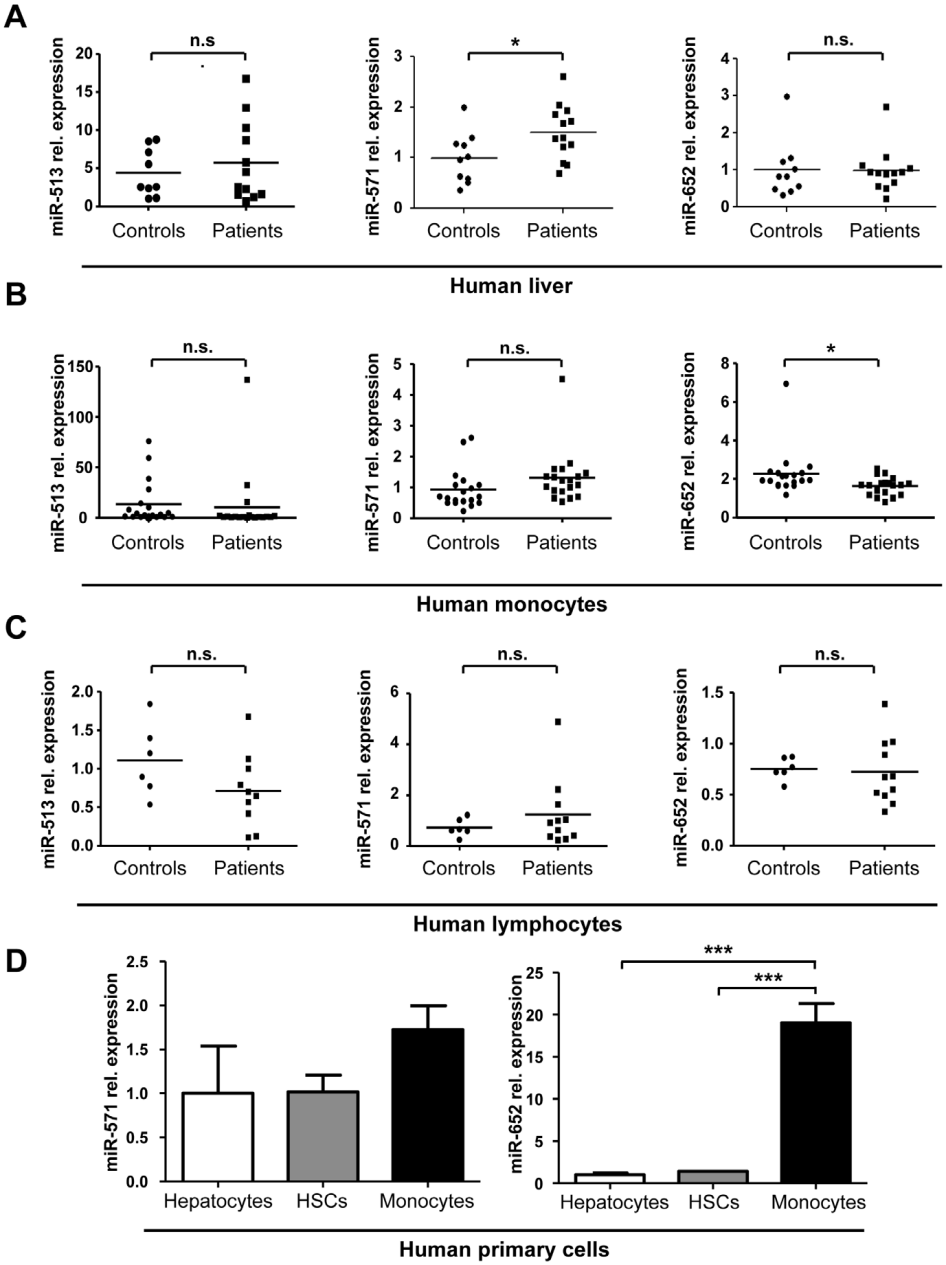
Serum levels of miR-571 and miR-652 reflect their specific regulation in distinct organs and cell compartments involved in the pathogenesis of liver cirrhosis. (A) Expression of miR-513-3p, miR-571 and miR-652 was analyzed by qPCR in liver samples of patients with liver cirrhosis (n = 13) and livers from patients without chronic liver disease (n = 10) as control. (B) Expression of miR-513-3p, miR-571 and miR-652 was analyzed by qPCR in MACS-sorted monocytes of patients with liver cirrhosis (n = 19) and healthy donors (n = 19) as control. (C) Expression of miR-513-3p, miR-571 and miR-652 was analyzed by qPCR in lymphocytes of patients with liver cirrhosis (n = 11) and healthy donors (n = 6) as control. Results are depicted as dot plot with each dot representing one individual patient. The line represents the mean of relative expression. (D) qPCR analysis of miR-571 and miR-652 expression in human primary hepatocytes, HSCs and monocytes, respectively. Results are depicted as mean, error bars denote SEM. *P<0.05, ***P<0.001.

To test the hypothesis that these miRNAs are differentially regulated within distinct compartments, we analyzed primary human liver tissue from cirrhotic patients (Data S1) and also isolated primary circulating monocytes as well as lymphocytes from patients with liver cirrhosis and healthy controls at high purities using magnetic bead separation techniques (Data S1). Interestingly, only levels of miR-571, but not of miR-652 or miR-513-3p, were concordantly regulated between serum and liver tissue from cirrhosis patients compared to healthy controls (Figure 4 A), suggesting that the liver is the primary source of serum miR-571 in cirrhosis patients. In contrast and concordant with its serum-specific regulation, miR-652 was significantly down-regulated in circulating monocytes isolated from patients compared to controls (Figure 4 B), while none of the three miRNAs were significantly dysregulated in the lymphocyte compartment (Figure 4 C).

In order to elucidate which hepatic cell population involved in liver cirrhosis likely contributes to miR-571 regulation in liver and serum of cirrhotic patients, we compared cell type-specific expression levels of these miRNAs in isolated primary human hepatocytes, hepatic stellate cells and monocytes/macrophages (Figure 4 D). Interestingly, miR-571 was expressed at similar levels in all of these cell compartments. In contrast, expression of miR-652 was restricted to monocytes/macrophages, indicating primary functions in regulating innate immune responses. Altogether, these data indicate that alterations of serum levels of miR-571 and miR-652 in liver cirrhosis patients reflect distinct regulatory mechanisms in different cell compartments involved in this disease, namely the liver and monocytes.

### miR-571 and miR-652 integrate pro-fibrotic and inflammatory signals in human HSCs and monocytes

**Figure 5:**
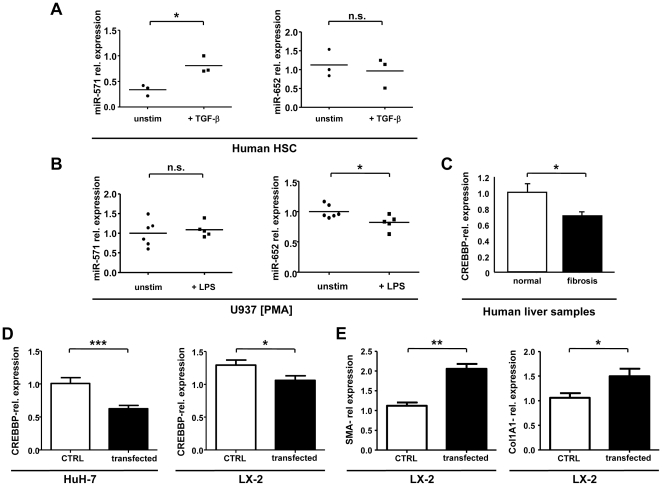
miR-571 and miR-652 integrate pro-fibrotic and inflammatory signals in human HSCs and monocytes. (A) Expression of miR-571 and miR-652 in primary human HSCs in response to stimulation with TGF-β for 48 h was measured by qPCR. Results are depicted as dot plot with each dot representing one individual patient. The line represents the mean of relative expression. (B) We stimulated U937 cells with PMA into a monocytic differentiation. miR-571 and miR-652 expression levels in response to stimulation with LPS for 48 h were measured by qPCR. Results are depicted as dot plot with each dot representing one individual patient. The line represents the mean of relative expression. (C) Expression of CREBBP was analyzed by qPCR in liver samples of patients with liver cirrhosis (n = 13) and non-liver diseased donors (n = 10) as control. Results are depicted as mean, error bars denote SEM. (D) HuH-7 cells and LX-2 cells were transfected with indicated amounts of miR-571 mimic. Expression of CREBBP was determined after two days of transfection by qPCR. Results are depicted as mean, error bars denote SEM. (E) Activation of stellate cells was determined by qPCR-based analysis of SMA- and Col1A1-expression in LX-2 cells. **P<0.01, ***P<0.001.

Based on the prominent up-regulation of hepatic and circulating miR-571 in cirrhotic patients, we hypothesized that miR-571 might be centrally involved in cellular responses to profibrogenic signals. The most relevant collagen-producing cell types in liver fibrosis are HSCs, and the cytokine TGF-β is one of the key profibrogenic mediators [32], [33]. When primary human HSCs, isolated from cirrhotic liver explants, were stimulated with TGF-β, miR-571 was significantly up-regulated (Figure 5 A). On the other hand, miR-652 did not show a specific regulation upon TGF-β stimulation. However, stimulation of monocytic U937 cells with the proinflammatory ligand bacterial lipopolysaccharide (LPS) led to a significant down-regulation of miR-652, while miR-571 remained unaffected (Figure 5 B). These data support that miR-571 is involved in the regulation of hepatic fibrogenesis, while miR-652 is part of an inflammatory signaling network accompanying the pathophysiological processes during liver cirrhosis in monocytes/macrophages.

To our knowledge, there is currently no published information on possible functions and putative target genes of these respective miRNAs available. To get a first insight into their function, we performed a broad *in silico*- target screen for putative miR-571 targets, the miRNA with the most prominent regulation in our analysis, by using two different miRNA-target prediction algorithms. Among others, we identified CREB-binding protein (CREBBP) as a possible target gene for miR-571 (Data S1), a gene involved in general transcriptional regulation previously associated with the regulation and pathogenesis of liver fibrosis [34], [35]. In line with up-regulation of miR-571, CREBBP was down-regulated in liver tissue from cirrhosis patients compared with healthy controls (Figure 5 C). Moreover, transfection of HuH-7 and LX-2 cells, cell lines representing human hepatocytes or HSC, respectively, with miR-571 led to a moderate, but significant down-regulation of CREBBP on RNA level (Figure 5 D), suggesting that CREBBP might be one possible target gene mediating miR-571-dependent effects in the regulation of liver cirrhosis. Finally, up-regulation of miR-571 leads to a moderate up-regulation of SMA and Col1A1 in LX-2 cells, indicating that miR-571 might be involved in activation of stellate cells during fibrogenesis (Figure 5 E).

## Discussion

While previous screening approaches to study the role of miRNAs in liver fibrosis/cirrhosis focused on their regulation in liver tissue or hepatic stellate cells [10], [11], [12], in the present study we performed a systematic array approach on serum samples from liver cirrhosis patients. By this approach, we identified a set of three miRNAs, miR-513-3p, miR-571 and miR-652, which to our knowledge have previously not been characterized in liver cirrhosis nor in any other physiological or pathological condition in humans or rodents. The key prerequisite for their identification was the significant alteration in serum even in the relatively small collective of samples included in the array analysis, which was then confirmed within the larger cohort of chronic liver disease patients. To our opinion, this approach thus represents a successful methodological alternative to previous studies, which mainly addressed regulations of pre-defined, single miRNAs in serum [10]. Moreover, our array approach included patients with alcoholic and hepatitis C-related liver disease, enabling us to identify miRNAs with putative etiology-independent roles in hepatic cirrhosis. From these three miRNAs, miR-571 not only identified most reliably patients with chronic liver diseases, but was also closely associated with cirrhosis progression, underlining its potential as a novel serum biomarker. Therefore, further evaluation in larger prospective trials is warranted to assess its potential diagnostic or prognostic value in patients with hepatic fibrosis or cirrhosis.

Serum parameters in general can show certain variability, and the reproducibility of serum tests depends on the nature of the marker itself as well as the standardized handling and preparation of the sample. While protein based markers are more or less prone to degradation [20], it has been consistently shown that serum miRNAs remain stable after being subjected to severe conditions that would normally degrade most RNAs, such as boiling, very low or high pH levels, extended storage, and 10 freeze–thaw cycles [36]. Moreover, specimen collection, processing, and purification of miRNA were strictly standardized in the present study in order to avoid any bias (see Materials and Methods). In line with numerous previously published studies [6], [17], [19], [20], we used spiked-in RNAs that were added into the serum sample before miRNA extraction for normalization of miRNA serum levels. This method is preferred by many groups to other normalization methods. As such, it was suggested that frequently used reference genes like U6 small nuclear RNA (RNU6B) and 5S ribosomal RNA show a less stable expression than others and are easily degraded in serum samples [37], [38]. Furthermore, levels of certain miRNAs (miR-142-3p and miR-16) that were claimed to be evenly expressed in patient and control serum samples in some studies were found to be significantly altered in others [17], [39]. The fact that by using spiked-in RNA for normalization we could show a similar regulation of certain miRNAs (miR-21, miR-34a, miR-122) [17], [27], [28] as demonstrated previously in other cohorts of cirrhotics (Figure S4) as well as our confirmatory experiments in a second, independent cohort of liver cirrhosis patients (Figure S4) strongly support the robustness of the de-regulation of the newly identified miRNAs in chronic liver disease.

The key finding of this study was a striking concordance between miRNA-levels in the serum and their specific regulations in different cellular compartments involved in the pathogenesis of chronic liver disease, raising the yet unanswered question which molecular processes underlie regulation of miRNA serum levels and if serum-miRNAs might withhold a specific function. On one hand, increased levels of miRNAs such as miR-513-3p or miR-571 might reflect an epiphenomenon resulting from increased hepatic cell death in chronic liver disease, a hypothesis that cannot be ruled out on basis of our data. However, some recent data have provided evidence that some miRNAs are not only passively released but in turn actively secreted from cells and integrated into vesicles, potentially serving as messengers to influence gene transcription in other cells [29], [30]. The specific up-regulation of miR-571 in HSC, the main matrix-producing cell compartment activated during fibrogenesis, in response to TGF-β, the most prominent pro-fibrogenic cytokine [32], supports a contribution of these cells to changes in miR-571 serum levels. Given the vast amount of potential miR-571-target genes, among those CREBBP, a general modulator of gene transcription [40], it is conceivable that miR-571 is released from certain hepatic cells in an active process to modulate transcriptional programs in other cells or cell compartments involved in liver cirrhosis. Interestingly, we previously demonstrated a similarly concordant regulation between liver tissue and serum levels in liver cirrhosis for miR-29 [10]. In contrast, miR-221, which is up-regulated in livers of cirrhosis patients [26], showed a down-regulation in the serum in our array analysis (see Figure 1 A), which was confirmed as significant by qPCR in our large collective (Figure S2). These data suggest that different molecular processes are connecting serum levels of distinct miRNA with their tissue-specific regulation in the liver. However, the quantitative relations between alterations in intracellular miRNA concentrations and serum miRNA levels are not known.

miR-652 represented another strongly regulated miRNA in patients with chronic liver disease, on which further experiments revealed interesting differences to miR-571. Most strikingly, miR-652 showed a significant down-regulation in patients with chronic liver disease, correlating with its regulation in circulating monocytes rather than parenchymal or resident liver cells. The selective expression of miR-652 in innate immune cells supports the hypothesis that indeed this cellular down-regulation played a causative role for decreased serum levels in the patient group. The finding that miR-652 levels integrated proinflammatory stimuli like LPS suggests that this miRNA is involved in the mediation of inflammatory signals in immune cells. These mechanisms are likely not liver-specific, but might represent a general innate immune response mechanism present in many chronic inflammatory conditions such as hepatic fibrosis. In line with this hypothesis, miR-652 showed a high predictive value for the presence of chronic liver disease but did not correlate with the stage of cirrhosis. Therefore, next to further functional characterizations in monocytes/macrophages, it might be interesting to test serum levels and the predictive value of miR-652 in other acute and chronic inflammatory disease states, e.g. rheumatoid arthritis, inflammatory bowel disease or sepsis.

In conclusion, using a systematic array approach on serum samples from patients with chronic liver disease, we identified etiology-independent alterations of serum levels of miR-513-3p, miR-571 and miR-652, three previously uncharacterized miRNAs. Moreover, we demonstrated that miR571- and miR-652- serum levels in patients with chronic liver disease reflect their putative roles in the mediation of fibrogenic and inflammatory processes in distinct cellular compartments involved in the pathogenesis of liver cirrhosis. These results might lead to the identification of novel regulatory networks in different cellular compartments as a basis for future miRNA-based therapeutic strategies for the treatment of chronic liver disease and liver cirrhosis.

## Supporting Information

Data S1 **Overview of supplementary data and supplementary tables.**
(DOC)

Figure S1 **SV40 and U6 were used for normalization of PCR based analysis.** (A) SV40 levels, used for normalization in further experiments, were determined by qPCR in sera of patients with liver cirrhosis and controls. (B) U6 expression, used for normalization in further experiments, were determined by qPCR in livers samples of patients with liver cirrhosis and controls.
(TIF)

Figure S2 **Confirmation of the array based results by using qPCR.** (A) Levels of miR-513-3p, miR-571 and miR-652 were analyzed by qPCR in the samples used for the initial array analysis in order to confirm the array based results. (B) miR-29a, miR-221 and miR-10a were analyzed by qPCR in the samples used for the initial array analysis in order to further confirm the array based results by extending this analysis on other miRNAs. (C) Serum levels of miR-29a, miR-221 and miR-10a were analyzed in the large collective of patients with liver cirrhosis and controls, revealing comparable results to the analysis in the smaller collective used for array analysis. *P<0.05, ***P<0.001.
(TIF)

Figure S3 **Confirmation of alterations of serum levels of miR-513-3p, miR-571 and miR-652 in a second cohort of patients.** Serum level of miR-513-3p, miR-571 and miR-652 were analyzed in a second collective of patients with chronic liver disease and patients without chronic liver disease (n = 13 each) in order to confirm that the alterations remain stable when patients instead of healthy individuals were used as control. *P<0.05.
(TIF)

Figure S4 **Validation of the array- and qPCR based data.** (A) Microarray analysis for miRNA levels was performed using RNA extracts from serum of four healthy subjects as control and patients with liver cirrhosis of different etiologies and stages of disease (for detailed clinical parameters see Data S1). Three miRNAs previously published in the context of liver cirrhosis are depicted to confirm the reliability of the array based results. (B) Serum levels of miR-21, miR-34a and miR-122 were analyzed in the large collective of patients with liver cirrhosis and controls, revealing comparable alterations to previously published results. **P<0.01, ***P<0.001.
(TIF)

Figure S5 **Analysis of miR-140 and miR-340 in the serum of patients with liver cirrhosis.** (A) Serum levels of miR-140 and miR-340 in the patients with alcoholic liver cirrhosis (Child-Pugh A and Child-Pugh C) were analyzed by qPCR. Results are depicted as box plot. The thick line represents median of relative expression. (B) ROC curve analysis displaying the diagnostic power of selected miRNAs in predicting advanced stage of liver cirrhosis.
(TIF)
