# Supplementary material for: Micro-RNA Profiling in Human Serum Reveals Compartment-Specific Roles of miR-571 and miR-652 in Liver Cirrhosis
Source: PLoS One. 2012 Mar 7;7(3):e32999. doi: 10.1371/journal.pone.0032999 (PMC3296762; doi:10.1371/journal.pone.0032999)
Supplement: Data S1 — Overview of supplementary data and supplementary tables. (DOC) [file pone.0032999.s001.doc]

**Supplementary information**

**Systematic micro-RNA profiling in human serum reveals entity-independent, compartment-specific roles of miR-571 and miR-652 as biomarkers in liver cirrhosis**

Christoph Roderburg, Tobias Mollnow, Brenda Bongaertz, Natalia Elfimova, David Vargas Cardenas, Katharina Berger, Henning Zimmermann, Alexander Koch, Mihael Vucur, Claus Hellerbrand, Margarete Odenthal, Frank Tacke, Christian Trautwein, Tom Luedde

**Inventory of Supporting Information**

**Supplementary Data set**

Suppl. figure 1: Validation of SV-40 and U6 as internal controls for miRNA-qPCR

Suppl. figure 2: Validation of the micro-array

Suppl. figure 3: Analysis of a second cohort of patients

Suppl. figure 4: Analysis of miR-21, miR-34a, miR-122 in patients´ serum

Suppl. figure 5: miRNA as discriminators between early and advanced cirrhosis

Suppl. table 1: Clinical data of patients with liver cirrhosis.

Suppl. table 2: Correlation of laboratory parameters with miRNA serum levels.

Suppl. table 3: Clinical data of patients and controls.

Suppl. table 4: Clinical data of patients with liver cirrhosis.

Suppl. table 5: Potential targets of miR-513-3p, miR-571 and miR-652

**Supplementary Materials and Methods**

Quantitative realtime-PCR- cell culture, stimulation and transfection- *in silico* analysis of potential miRNA target genes- statistics

**Supplementary References**

1 Supplemental Reference

**Supplementary Data Set**

**Supplementary tables:**

**Supplementary table** 1

|  | **Patient characteristics (array analysis)** | | | | |
| --- | --- | --- | --- | --- | --- |
| **Patient #** | **Age** | **Sex** | **Etiology** | **Staging** | **MELD** |
| 1 | 73 | w | EtOH | Child A | 11 |
| 2 | 68 | m | EtOH | Child A | 6 |
| 3 | 37 | w | EtOH | Child A | 8 |
| 4 | 62 | m | EtOH | Child A | 11 |
| 5 | 47 | m | EtOH | Child C | 21 |
| 6 | 72 | m | EtOH | Child C | 16 |
| 7 | 66 | m | EtOH | Child C | 19 |
| 8 | 73 | w | EtOH | Child C | 19 |
| 9 | 60 | m | viral | Child A | 7 |
| 10 | 57 | w | viral | Child A | 10 |
| 11 | 60 | m | viral | Child A | 6 |
| 12 | 55 | m | viral | Child A | 7 |

**Supplementary table 1: Clinical data of patients with liver cirrhosis.** Clinical data of patients with liver cirrhosis that were used for array analysis of miRNA (figure 1). Presence of liver cirrhosis was performed by two independent and blinded liver pathologists. EtOH: Ethanol abuse; PSC: primary sclerosing cholangitis; viral etiology included both HBV and HCV.

**Supplementary t**able 2

|  | **miR-513-3p** | | **miR-571** | | **miR-652** | |
| --- | --- | --- | --- | --- | --- | --- |
|  | r | p | r | p | r | p |
| **Creatinine** | 0.134 | 0.334 | 0.115 | 0.387 | 0.023 | 0.858 |
| **Albumin** | - 0.092 | 0.510 | - 0.359 | **0.005** | 0.137 | 0.276 |
| **Child-Points** | 0.239 | 0.099 | 0.274 | **0.049** | - 0.095 | 0.483 |
| **GLDH** | - 0.048 | 0.741 | - 0.292 | **0.032** | 0.021 | 0.872 |
| **INR** | - 0.042 | 0.763 | 0.288 | **0.027** | - 0.222 | 0.076 |
| **PCHE** | - 0.017 | 0.903 | - 0.328 | **0.011** | - 0.148 | 0.239 |
| **Quick** | 0.050 | 0.721 | - 0.300 | **0.021** | 0.243 | 0.051 |

**Supplementary table 2: Correlation of laboratory parameters with miRNA serum levels.** Correlation of serum level of each individual miRNA with important laboratory parameters. r: correlation coefficient; p: p-value.

**Supplementary table** 3

|  | **Patient characteristics** | | |
| --- | --- | --- | --- |
| **Patient #** | **Etiology** | **Staging** | **Inflammation** |
| 1 | PSC | 4 | 2 – 3 |
| 2 | viral | 4 | 2 – 3 |
| 3 | viral | 4 | 3 |
| 4 | viral | 4 | 3 |
| 5 | PSC | 3 | 2 |
| 6 | EtOH | 4 | 2 – 3 |
| 7 | EtOH | 4 | 2 |
| 8 | viral | 2 | 2 |
| 9 | viral | 4 | 2 |
| 10 | viral | 4 | 2 – 3 |
| 11 | viral | 4 | 2 – 3 |
| 12 | viral | 3 | 3 |
| 13 | EtOH | 4 | 2 – 3 |

**Supplementary table 3: Clinical data of patients with liver cirrhosis.** Clinical data of patients with liver cirrhosis that were used for qRT-PCR analysis of miRNA expression (figure 4A). Grading and staging were performed by two independent and blinded liver pathologists. The desmet score was used for grading and staging. EtOH: Ethanol abuse; PSC: primary sclerosing cholangitis; viral etiology included both HBV and HCV.

**Supplementary t**able 4

|  |  |  | **Child-Pugh stages of liver cirrhosis** | | | |
| --- | --- | --- | --- | --- | --- | --- |
|  | **controls** | **all patients** | **no cirrhosis** | **Child A** | **Child B** | **Child C** |
| **Monocytes** |  |  |  |  |  |  |
| [n (%)] | 20 | 20 |  |  |  | 20 (100 %) |
| Gender (male/female) [n] | 16/4 | 16/4 |  |  |  | 16/4 |
| Age (mean and range in years) | 41 (24-65) | 56 (38-76) |  |  |  | 56 (38-76) |
| Etiology of liver disease |  |  |  |  |  |  |
| Viral hepatitis [n (%)] |  | 1 (5 %) |  |  |  |  |
| Biliary [n (%)] |  | 0 (0 %) |  |  |  |  |
| Alcohol [n (%)] |  | 19 (95 %) |  |  |  |  |
| Other [n (%)] |  | 0 (0 %) |  |  |  |  |
|  |  |  |  |  |  |  |
| **Lymphocytes** | |  |  |  |  |  |
| [n (%)] | 5 | 11 | 1 (9 %) | 2 (18 %) | 4 (36 %) | 4 (36 %) |
| Gender (male/female) [n] | 2/3 | 8/3 | 1/0 | 1/1 | 3/1 | 3/1 |
| Age (mean and range in years) | 51 (36-65) | 54 (38-69) | 55 | 58 (50-65) | 60 (40-69) | 47 (38-57) |
| Etiology of liver disease |  |  |  |  |  |  |
| Viral hepatitis [n (%)] |  | 4 (36 %) | 1 (100 %) | 1 (50 %) | 2 (50 %) | 0 (0 %) |
| Biliary [n (%)] |  | 0 (0 %) | 0 (0 %) | 0 (0 %) | 0 (0 %) | 0 (0 %) |
| Alcohol [n (%)] |  | 7 (64 %) | 0 (0 %) | 1 (50 %) | 2 (50 %) | 4 (100 %) |
| Other [n (%)] |  | 0 (0 %) | 0 (0 %) | 0 (0 %) | 0 (0 %) | 0 (0 %) |

**Supplementary table 4: Clinical data of patients and controls.** Monocytes and lymphocytes were isolated from patients and controls for qRT-PCR analysis of miRNA expression (figure 4B, C). Viral hepatitis included only HCV.

| **miR-513-3p** |  |
| --- | --- |
| **Target Gene** | **Gene name** |
| [SLC7A11](http://www.ncbi.nlm.nih.gov/sites/entrez?Db=gene&Cmd=ShowDetailView&TermToSearch=23657) | solute carrier family 7, (cationic amino acid transporter, y+ system) member 11 |
| [ITGA4](http://www.ncbi.nlm.nih.gov/sites/entrez?Db=gene&Cmd=ShowDetailView&TermToSearch=3676) | integrin, alpha 4 (antigen CD49D, alpha 4 subunit of VLA-4 receptor) |
| [SFRS12IP1](http://www.ncbi.nlm.nih.gov/sites/entrez?Db=gene&Cmd=ShowDetailView&TermToSearch=285672) | SFRS12-interacting protein 1 |
| [PHF20L1](http://www.ncbi.nlm.nih.gov/sites/entrez?Db=gene&Cmd=ShowDetailView&TermToSearch=51105) | PHD finger protein 20-like 1 |
| [TRDMT1](http://www.ncbi.nlm.nih.gov/sites/entrez?Db=gene&Cmd=ShowDetailView&TermToSearch=1787) | tRNA aspartic acid methyltransferase 1 |
| [ELAVL2](http://www.ncbi.nlm.nih.gov/sites/entrez?Db=gene&Cmd=ShowDetailView&TermToSearch=1993) | ELAV (embryonic lethal, abnormal vision, Drosophila)-like 2 (Hu antigen B) |
| [EXOC5](http://www.ncbi.nlm.nih.gov/sites/entrez?Db=gene&Cmd=ShowDetailView&TermToSearch=10640) | exocyst complex component 5 |
| [ZBTB41](http://www.ncbi.nlm.nih.gov/sites/entrez?Db=gene&Cmd=ShowDetailView&TermToSearch=360023) | zinc finger and BTB domain containing 41 |
| [CNOT6L](http://www.ncbi.nlm.nih.gov/sites/entrez?Db=gene&Cmd=ShowDetailView&TermToSearch=246175) | CCR4-NOT transcription complex, subunit 6-like |
| [ZNF148](http://www.ncbi.nlm.nih.gov/sites/entrez?Db=gene&Cmd=ShowDetailView&TermToSearch=7707) | zinc finger protein 148 |
| [SLC2A13](http://www.ncbi.nlm.nih.gov/sites/entrez?Db=gene&Cmd=ShowDetailView&TermToSearch=114134) | solute carrier family 2 (facilitated glucose transporter), member 13 |
| [LHCGR](http://www.ncbi.nlm.nih.gov/sites/entrez?Db=gene&Cmd=ShowDetailView&TermToSearch=3973) | luteinizing hormone/choriogonadotropin receptor |
| [RYBP](http://www.ncbi.nlm.nih.gov/sites/entrez?Db=gene&Cmd=ShowDetailView&TermToSearch=23429) | RING1 and YY1 binding protein |
| [AFF4](http://www.ncbi.nlm.nih.gov/sites/entrez?Db=gene&Cmd=ShowDetailView&TermToSearch=27125) | AF4/FMR2 family, member 4 |
| [TMF1](http://www.ncbi.nlm.nih.gov/sites/entrez?Db=gene&Cmd=ShowDetailView&TermToSearch=7110) | TATA element modulatory factor 1 |
| [HMGB1](http://www.ncbi.nlm.nih.gov/sites/entrez?Db=gene&Cmd=ShowDetailView&TermToSearch=3146) | high-mobility group box 1 |
| [ZBTB8](http://www.ncbi.nlm.nih.gov/sites/entrez?Db=gene&Cmd=ShowDetailView&TermToSearch=653121) | zinc finger and BTB domain containing 8 |
| [LRP6](http://www.ncbi.nlm.nih.gov/sites/entrez?Db=gene&Cmd=ShowDetailView&TermToSearch=4040) | low density lipoprotein receptor-related protein 6 |
| [UBE2K](http://www.ncbi.nlm.nih.gov/sites/entrez?Db=gene&Cmd=ShowDetailView&TermToSearch=3093) | ubiquitin-conjugating enzyme E2K (UBC1 homolog, yeast) |
| [ITGB8](http://www.ncbi.nlm.nih.gov/sites/entrez?Db=gene&Cmd=ShowDetailView&TermToSearch=3696) | integrin, beta 8 |
| [SLC26A2](http://www.ncbi.nlm.nih.gov/sites/entrez?Db=gene&Cmd=ShowDetailView&TermToSearch=1836) | solute carrier family 26 (sulfate transporter), member 2 |
| [PTP4A1](http://www.ncbi.nlm.nih.gov/sites/entrez?Db=gene&Cmd=ShowDetailView&TermToSearch=7803) | protein tyrosine phosphatase type IVA, member 1 |
| [NUS1](http://www.ncbi.nlm.nih.gov/sites/entrez?Db=gene&Cmd=ShowDetailView&TermToSearch=116150) | nuclear undecaprenyl pyrophosphate synthase 1 homolog (S. cerevisiae) |
| [TBC1D12](http://www.ncbi.nlm.nih.gov/sites/entrez?Db=gene&Cmd=ShowDetailView&TermToSearch=23232) | TBC1 domain family, member 12 |
| [SR140](http://www.ncbi.nlm.nih.gov/sites/entrez?Db=gene&Cmd=ShowDetailView&TermToSearch=23350) | U2-associated SR140 protein |
| [SNX15](http://www.ncbi.nlm.nih.gov/sites/entrez?Db=gene&Cmd=ShowDetailView&TermToSearch=29907) | sorting nexin 15 |
| [GCNT1](http://www.ncbi.nlm.nih.gov/sites/entrez?Db=gene&Cmd=ShowDetailView&TermToSearch=2650) | glucosaminyl (N-acetyl) transferase 1, core 2 (beta-1,6-N-acetylglucosaminyltransferase) |
| [SNX14](http://www.ncbi.nlm.nih.gov/sites/entrez?Db=gene&Cmd=ShowDetailView&TermToSearch=57231) | sorting nexin 14 |
| [C13orf1](http://www.ncbi.nlm.nih.gov/sites/entrez?Db=gene&Cmd=ShowDetailView&TermToSearch=57213) | chromosome 13 open reading frame 1 |
| [NUFIP2](http://www.ncbi.nlm.nih.gov/sites/entrez?Db=gene&Cmd=ShowDetailView&TermToSearch=57532) | nuclear fragile X mental retardation protein interacting protein 2 |
| [C5orf30](http://www.ncbi.nlm.nih.gov/sites/entrez?Db=gene&Cmd=ShowDetailView&TermToSearch=90355) | chromosome 5 open reading frame 30 |
| [C4orf46](http://www.ncbi.nlm.nih.gov/sites/entrez?Db=gene&Cmd=ShowDetailView&TermToSearch=201725) | chromosome 4 open reading frame 46 |
| [DNER](http://www.ncbi.nlm.nih.gov/sites/entrez?Db=gene&Cmd=ShowDetailView&TermToSearch=92737) | delta/notch-like EGF repeat containing |
| [FBXO8](http://www.ncbi.nlm.nih.gov/sites/entrez?Db=gene&Cmd=ShowDetailView&TermToSearch=26269) | F-box protein 8 |
| [PCDH11X](http://www.ncbi.nlm.nih.gov/sites/entrez?Db=gene&Cmd=ShowDetailView&TermToSearch=27328) | protocadherin 11 X-linked |
| [TSC22D1](http://www.ncbi.nlm.nih.gov/sites/entrez?Db=gene&Cmd=ShowDetailView&TermToSearch=8848) | TSC22 domain family, member 1 |
| [FEM1C](http://www.ncbi.nlm.nih.gov/sites/entrez?Db=gene&Cmd=ShowDetailView&TermToSearch=56929) | fem-1 homolog c (C. elegans) |
| [MED13](http://www.ncbi.nlm.nih.gov/sites/entrez?Db=gene&Cmd=ShowDetailView&TermToSearch=9969) | mediator complex subunit 13 |
| [STXBP5L](http://www.ncbi.nlm.nih.gov/sites/entrez?Db=gene&Cmd=ShowDetailView&TermToSearch=9515) | syntaxin binding protein 5-like |
| [CUL3](http://www.ncbi.nlm.nih.gov/sites/entrez?Db=gene&Cmd=ShowDetailView&TermToSearch=8452) | cullin 3 |
| [ZFAND5](http://www.ncbi.nlm.nih.gov/sites/entrez?Db=gene&Cmd=ShowDetailView&TermToSearch=7763) | zinc finger, AN1-type domain 5 |
| [KIAA0101](http://www.ncbi.nlm.nih.gov/sites/entrez?Db=gene&Cmd=ShowDetailView&TermToSearch=9768) | KIAA0101 |
| [NCOA1](http://www.ncbi.nlm.nih.gov/sites/entrez?Db=gene&Cmd=ShowDetailView&TermToSearch=8648) | nuclear receptor coactivator 1 |
| [LIN54](http://www.ncbi.nlm.nih.gov/sites/entrez?Db=gene&Cmd=ShowDetailView&TermToSearch=132660) | lin-54 homolog (C. elegans) |
| [C4orf32](http://www.ncbi.nlm.nih.gov/sites/entrez?Db=gene&Cmd=ShowDetailView&TermToSearch=132720) | chromosome 4 open reading frame 32 |
| [FBXL3](http://www.ncbi.nlm.nih.gov/sites/entrez?Db=gene&Cmd=ShowDetailView&TermToSearch=26224) | F-box and leucine-rich repeat protein 3 |
| [IYD](http://www.ncbi.nlm.nih.gov/sites/entrez?Db=gene&Cmd=ShowDetailView&TermToSearch=389434) | iodotyrosine deiodinase |
| [CREBZF](http://www.ncbi.nlm.nih.gov/sites/entrez?Db=gene&Cmd=ShowDetailView&TermToSearch=58487) | CREB/ATF bZIP transcription factor |
| [XIAP](http://www.ncbi.nlm.nih.gov/sites/entrez?Db=gene&Cmd=ShowDetailView&TermToSearch=331) | X-linked inhibitor of apoptosis |
| [YWHAZ](http://www.ncbi.nlm.nih.gov/sites/entrez?Db=gene&Cmd=ShowDetailView&TermToSearch=7534) | tyrosine 3-monooxygenase/tryptophan 5-monooxygenase activation protein, zeta polypeptide |
| [FAM175B](http://www.ncbi.nlm.nih.gov/sites/entrez?Db=gene&Cmd=ShowDetailView&TermToSearch=23172) | family with sequence similarity 175, member B |
| [LARP4](http://www.ncbi.nlm.nih.gov/sites/entrez?Db=gene&Cmd=ShowDetailView&TermToSearch=113251) | La ribonucleoprotein domain family, member 4 |
| [NOTCH2](http://www.ncbi.nlm.nih.gov/sites/entrez?Db=gene&Cmd=ShowDetailView&TermToSearch=4853) | Notch homolog 2 (Drosophila) |
| [C4orf18](http://www.ncbi.nlm.nih.gov/sites/entrez?Db=gene&Cmd=ShowDetailView&TermToSearch=51313) | chromosome 4 open reading frame 18 |
| [PFKFB3](http://www.ncbi.nlm.nih.gov/sites/entrez?Db=gene&Cmd=ShowDetailView&TermToSearch=5209) | 6-phosphofructo-2-kinase/fructose-2,6-biphosphatase 3 |
| [DSEL](http://www.ncbi.nlm.nih.gov/sites/entrez?Db=gene&Cmd=ShowDetailView&TermToSearch=92126) | dermatan sulfate epimerase-like |
| [DICER1](http://www.ncbi.nlm.nih.gov/sites/entrez?Db=gene&Cmd=ShowDetailView&TermToSearch=23405) | dicer 1, ribonuclease type III |
| [PCTK2](http://www.ncbi.nlm.nih.gov/sites/entrez?Db=gene&Cmd=ShowDetailView&TermToSearch=5128) | PCTAIRE protein kinase 2 |
| [CDC2L6](http://www.ncbi.nlm.nih.gov/sites/entrez?Db=gene&Cmd=ShowDetailView&TermToSearch=23097) | cell division cycle 2-like 6 (CDK8-like) |
| [GMFB](http://www.ncbi.nlm.nih.gov/sites/entrez?Db=gene&Cmd=ShowDetailView&TermToSearch=2764) | glia maturation factor, beta |
| [SSX2IP](http://www.ncbi.nlm.nih.gov/sites/entrez?Db=gene&Cmd=ShowDetailView&TermToSearch=117178) | synovial sarcoma, X breakpoint 2 interacting protein |
| [GDA](http://www.ncbi.nlm.nih.gov/sites/entrez?Db=gene&Cmd=ShowDetailView&TermToSearch=9615) | guanine deaminase |
| [IDS](http://www.ncbi.nlm.nih.gov/sites/entrez?Db=gene&Cmd=ShowDetailView&TermToSearch=3423) | iduronate 2-sulfatase (Hunter syndrome) |
| [RC3H1](http://www.ncbi.nlm.nih.gov/sites/entrez?Db=gene&Cmd=ShowDetailView&TermToSearch=149041) | ring finger and CCCH-type zinc finger domains 1 |
| [SYPL1](http://www.ncbi.nlm.nih.gov/sites/entrez?Db=gene&Cmd=ShowDetailView&TermToSearch=6856) | synaptophysin-like 1 |
| [KCNE4](http://www.ncbi.nlm.nih.gov/sites/entrez?Db=gene&Cmd=ShowDetailView&TermToSearch=23704) | potassium voltage-gated channel, Isk-related family, member 4 |
| [SLC30A6](http://www.ncbi.nlm.nih.gov/sites/entrez?Db=gene&Cmd=ShowDetailView&TermToSearch=55676) | solute carrier family 30 (zinc transporter), member 6 |
| [SMARCAD1](http://www.ncbi.nlm.nih.gov/sites/entrez?Db=gene&Cmd=ShowDetailView&TermToSearch=56916) | SWI/SNF-related, matrix-associated actin-dependent regulator of chromatin, subfamily a, containing DEAD/H box 1 |
| [PPP1R9A](http://www.ncbi.nlm.nih.gov/sites/entrez?Db=gene&Cmd=ShowDetailView&TermToSearch=55607) | protein phosphatase 1, regulatory (inhibitor) subunit 9A |
| [ZNF654](http://www.ncbi.nlm.nih.gov/sites/entrez?Db=gene&Cmd=ShowDetailView&TermToSearch=55279) | zinc finger protein 654 |
| [MATR3](http://www.ncbi.nlm.nih.gov/sites/entrez?Db=gene&Cmd=ShowDetailView&TermToSearch=9782) | matrin 3 |
| [IKZF2](http://www.ncbi.nlm.nih.gov/sites/entrez?Db=gene&Cmd=ShowDetailView&TermToSearch=22807) | IKAROS family zinc finger 2 (Helios) |
| [MYO5B](http://www.ncbi.nlm.nih.gov/sites/entrez?Db=gene&Cmd=ShowDetailView&TermToSearch=4645) | myosin VB |
| [SP3](http://www.ncbi.nlm.nih.gov/sites/entrez?Db=gene&Cmd=ShowDetailView&TermToSearch=6670) | Sp3 transcription factor |
| [MPPED2](http://www.ncbi.nlm.nih.gov/sites/entrez?Db=gene&Cmd=ShowDetailView&TermToSearch=744) | metallophosphoesterase domain containing 2 |
| [ESRRG](http://www.ncbi.nlm.nih.gov/sites/entrez?Db=gene&Cmd=ShowDetailView&TermToSearch=2104) | estrogen-related receptor gamma |
| [SLC5A12](http://www.ncbi.nlm.nih.gov/sites/entrez?Db=gene&Cmd=ShowDetailView&TermToSearch=159963) | solute carrier family 5 (sodium/glucose cotransporter), member 12 |
| [CCPG1](http://www.ncbi.nlm.nih.gov/sites/entrez?Db=gene&Cmd=ShowDetailView&TermToSearch=9236) | cell cycle progression 1 |
| [KIAA1712](http://www.ncbi.nlm.nih.gov/sites/entrez?Db=gene&Cmd=ShowDetailView&TermToSearch=80817) | KIAA1712 |
| [SC5DL](http://www.ncbi.nlm.nih.gov/sites/entrez?Db=gene&Cmd=ShowDetailView&TermToSearch=6309) | sterol-C5-desaturase (ERG3 delta-5-desaturase homolog, S. cerevisiae)-like |
| [USP31](http://www.ncbi.nlm.nih.gov/sites/entrez?Db=gene&Cmd=ShowDetailView&TermToSearch=57478) | ubiquitin specific peptidase 31 |
| [FMR1](http://www.ncbi.nlm.nih.gov/sites/entrez?Db=gene&Cmd=ShowDetailView&TermToSearch=2332) | fragile X mental retardation 1 |
| [DR1](http://www.ncbi.nlm.nih.gov/sites/entrez?Db=gene&Cmd=ShowDetailView&TermToSearch=1810) | down-regulator of transcription 1, TBP-binding (negative cofactor 2) |
| [LEPROT](http://www.ncbi.nlm.nih.gov/sites/entrez?Db=gene&Cmd=ShowDetailView&TermToSearch=54741) | leptin receptor overlapping transcript |
| [CUGBP2](http://www.ncbi.nlm.nih.gov/sites/entrez?Db=gene&Cmd=ShowDetailView&TermToSearch=10659) | CUG triplet repeat, RNA binding protein 2 |
| [CREG2](http://www.ncbi.nlm.nih.gov/sites/entrez?Db=gene&Cmd=ShowDetailView&TermToSearch=200407) | cellular repressor of E1A-stimulated genes 2 |
| [MAP9](http://www.ncbi.nlm.nih.gov/sites/entrez?Db=gene&Cmd=ShowDetailView&TermToSearch=79884) | microtubule-associated protein 9 |
| [TMTC1](http://www.ncbi.nlm.nih.gov/sites/entrez?Db=gene&Cmd=ShowDetailView&TermToSearch=83857) | transmembrane and tetratricopeptide repeat containing 1 |
| [ZADH2](http://www.ncbi.nlm.nih.gov/sites/entrez?Db=gene&Cmd=ShowDetailView&TermToSearch=284273) | zinc binding alcohol dehydrogenase domain containing 2 |
| [EFHA2](http://www.ncbi.nlm.nih.gov/sites/entrez?Db=gene&Cmd=ShowDetailView&TermToSearch=286097) | EF-hand domain family, member A2 |
| [TMEM1](http://www.ncbi.nlm.nih.gov/sites/entrez?Db=gene&Cmd=ShowDetailView&TermToSearch=7109) | transmembrane protein 1 |
| [UHRF1BP1](http://www.ncbi.nlm.nih.gov/sites/entrez?Db=gene&Cmd=ShowDetailView&TermToSearch=54887) | UHRF1 (ICBP90) binding protein 1 |
| [FAM133A](http://www.ncbi.nlm.nih.gov/sites/entrez?Db=gene&Cmd=ShowDetailView&TermToSearch=286499) | family with sequence similarity 133, member A |
| [KIAA2018](http://www.ncbi.nlm.nih.gov/sites/entrez?Db=gene&Cmd=ShowDetailView&TermToSearch=205717) | KIAA2018 |
| [PLCL1](http://www.ncbi.nlm.nih.gov/sites/entrez?Db=gene&Cmd=ShowDetailView&TermToSearch=5334) | phospholipase C-like 1 |
| [TMEM97](http://www.ncbi.nlm.nih.gov/sites/entrez?Db=gene&Cmd=ShowDetailView&TermToSearch=27346) | transmembrane protein 97 |
| [CTDSPL2](http://www.ncbi.nlm.nih.gov/sites/entrez?Db=gene&Cmd=ShowDetailView&TermToSearch=51496) | CTD (carboxy-terminal domain, RNA polymerase II, polypeptide A) small phosphatase like 2 |
| [PRDM16](http://www.ncbi.nlm.nih.gov/sites/entrez?Db=gene&Cmd=ShowDetailView&TermToSearch=63976) | PR domain containing 16 |
| [CSNK1A1](http://www.ncbi.nlm.nih.gov/sites/entrez?Db=gene&Cmd=ShowDetailView&TermToSearch=1452) | casein kinase 1, alpha 1 |
| [MARCKS](http://www.ncbi.nlm.nih.gov/sites/entrez?Db=gene&Cmd=ShowDetailView&TermToSearch=4082) | myristoylated alanine-rich protein kinase C substrate |
| [CALB1](http://www.ncbi.nlm.nih.gov/sites/entrez?Db=gene&Cmd=ShowDetailView&TermToSearch=793) | calbindin 1, 28kDa |
| [QKI](http://www.ncbi.nlm.nih.gov/sites/entrez?Db=gene&Cmd=ShowDetailView&TermToSearch=9444) | quaking homolog, KH domain RNA binding (mouse) |
| [TMEM200B](http://www.ncbi.nlm.nih.gov/sites/entrez?Db=gene&Cmd=ShowDetailView&TermToSearch=399474) | transmembrane protein 200B |
| [HOXC11](http://www.ncbi.nlm.nih.gov/sites/entrez?Db=gene&Cmd=ShowDetailView&TermToSearch=3227) | homeobox C11 |
| [TET2](http://www.ncbi.nlm.nih.gov/sites/entrez?Db=gene&Cmd=ShowDetailView&TermToSearch=54790) | tet oncogene family member 2 |
| [RANBP9](http://www.ncbi.nlm.nih.gov/sites/entrez?Db=gene&Cmd=ShowDetailView&TermToSearch=10048) | RAN binding protein 9 |
| [ZNF507](http://www.ncbi.nlm.nih.gov/sites/entrez?Db=gene&Cmd=ShowDetailView&TermToSearch=22847) | zinc finger protein 507 |
| [NFAT5](http://www.ncbi.nlm.nih.gov/sites/entrez?Db=gene&Cmd=ShowDetailView&TermToSearch=10725) | nuclear factor of activated T-cells 5, tonicity-responsive |
| [CLTC](http://www.ncbi.nlm.nih.gov/sites/entrez?Db=gene&Cmd=ShowDetailView&TermToSearch=1213) | clathrin, heavy chain (Hc) |
| [CCDC47](http://www.ncbi.nlm.nih.gov/sites/entrez?Db=gene&Cmd=ShowDetailView&TermToSearch=57003) | coiled-coil domain containing 47 |
| [TCF12](http://www.ncbi.nlm.nih.gov/sites/entrez?Db=gene&Cmd=ShowDetailView&TermToSearch=6938) | transcription factor 12 (HTF4, helix-loop-helix transcription factors 4) |
| [CHD7](http://www.ncbi.nlm.nih.gov/sites/entrez?Db=gene&Cmd=ShowDetailView&TermToSearch=55636) | chromodomain helicase DNA binding protein 7 |
| [LRRC4C](http://www.ncbi.nlm.nih.gov/sites/entrez?Db=gene&Cmd=ShowDetailView&TermToSearch=57689) | leucine rich repeat containing 4C |
| [ADAT2](http://www.ncbi.nlm.nih.gov/sites/entrez?Db=gene&Cmd=ShowDetailView&TermToSearch=134637) | adenosine deaminase, tRNA-specific 2, TAD2 homolog (S. cerevisiae) |
| [SMARCA1](http://www.ncbi.nlm.nih.gov/sites/entrez?Db=gene&Cmd=ShowDetailView&TermToSearch=6594) | SWI/SNF related, matrix associated, actin dependent regulator of chromatin, subfamily a, member 1 |
| [LRRC3B](http://www.ncbi.nlm.nih.gov/sites/entrez?Db=gene&Cmd=ShowDetailView&TermToSearch=116135) | leucine rich repeat containing 3B |
| [LPGAT1](http://www.ncbi.nlm.nih.gov/sites/entrez?Db=gene&Cmd=ShowDetailView&TermToSearch=9926) | lysophosphatidylglycerol acyltransferase 1 |
| [LUZP2](http://www.ncbi.nlm.nih.gov/sites/entrez?Db=gene&Cmd=ShowDetailView&TermToSearch=338645) | leucine zipper protein 2 |
| [KCNMA1](http://www.ncbi.nlm.nih.gov/sites/entrez?Db=gene&Cmd=ShowDetailView&TermToSearch=3778) | potassium large conductance calcium-activated channel, subfamily M, alpha member 1 |
| [NEUROG2](http://www.ncbi.nlm.nih.gov/sites/entrez?Db=gene&Cmd=ShowDetailView&TermToSearch=63973) | neurogenin 2 |
| [GHR](http://www.ncbi.nlm.nih.gov/sites/entrez?Db=gene&Cmd=ShowDetailView&TermToSearch=2690) | growth hormone receptor |
| [RP5-1022P6.2](http://www.ncbi.nlm.nih.gov/sites/entrez?Db=gene&Cmd=ShowDetailView&TermToSearch=56261) | hypothetical protein KIAA1434 |
| [NAB1](http://www.ncbi.nlm.nih.gov/sites/entrez?Db=gene&Cmd=ShowDetailView&TermToSearch=4664) | NGFI-A binding protein 1 (EGR1 binding protein 1) |
| [MAPK6](http://www.ncbi.nlm.nih.gov/sites/entrez?Db=gene&Cmd=ShowDetailView&TermToSearch=5597) | mitogen-activated protein kinase 6 |
| [LUC7L2](http://www.ncbi.nlm.nih.gov/sites/entrez?Db=gene&Cmd=ShowDetailView&TermToSearch=51631) | LUC7-like 2 (S. cerevisiae) |
| [TMCO3](http://www.ncbi.nlm.nih.gov/sites/entrez?Db=gene&Cmd=ShowDetailView&TermToSearch=55002) | transmembrane and coiled-coil domains 3 |
| [PHKB](http://www.ncbi.nlm.nih.gov/sites/entrez?Db=gene&Cmd=ShowDetailView&TermToSearch=5257) | phosphorylase kinase, beta |
| [MTMR9](http://www.ncbi.nlm.nih.gov/sites/entrez?Db=gene&Cmd=ShowDetailView&TermToSearch=66036) | myotubularin related protein 9 |
| [PITPNB](http://www.ncbi.nlm.nih.gov/sites/entrez?Db=gene&Cmd=ShowDetailView&TermToSearch=23760) | phosphatidylinositol transfer protein, beta |
| [PIGK](http://www.ncbi.nlm.nih.gov/sites/entrez?Db=gene&Cmd=ShowDetailView&TermToSearch=10026) | phosphatidylinositol glycan anchor biosynthesis, class K |
| [SLC5A3](http://www.ncbi.nlm.nih.gov/sites/entrez?Db=gene&Cmd=ShowDetailView&TermToSearch=6526) | solute carrier family 5 (inositol transporters), member 3 |
| [CASZ1](http://www.ncbi.nlm.nih.gov/sites/entrez?Db=gene&Cmd=ShowDetailView&TermToSearch=54897) | castor zinc finger 1 |
| [CERK](http://www.ncbi.nlm.nih.gov/sites/entrez?Db=gene&Cmd=ShowDetailView&TermToSearch=64781) | ceramide kinase |
| [JPH4](http://www.ncbi.nlm.nih.gov/sites/entrez?Db=gene&Cmd=ShowDetailView&TermToSearch=84502) | junctophilin 4 |
| [SNCA](http://www.ncbi.nlm.nih.gov/sites/entrez?Db=gene&Cmd=ShowDetailView&TermToSearch=6622) | synuclein, alpha (non A4 component of amyloid precursor) |
| [TRIM13](http://www.ncbi.nlm.nih.gov/sites/entrez?Db=gene&Cmd=ShowDetailView&TermToSearch=10206) | tripartite motif-containing 13 |
| [C9orf47](http://www.ncbi.nlm.nih.gov/sites/entrez?Db=gene&Cmd=ShowDetailView&TermToSearch=286223) | chromosome 9 open reading frame 47 |
| [UTX](http://www.ncbi.nlm.nih.gov/sites/entrez?Db=gene&Cmd=ShowDetailView&TermToSearch=7403) | ubiquitously transcribed tetratricopeptide repeat, X chromosome |
| [ANKRD17](http://www.ncbi.nlm.nih.gov/sites/entrez?Db=gene&Cmd=ShowDetailView&TermToSearch=26057) | ankyrin repeat domain 17 |
| [SPG20](http://www.ncbi.nlm.nih.gov/sites/entrez?Db=gene&Cmd=ShowDetailView&TermToSearch=23111) | spastic paraplegia 20 (Troyer syndrome) |
| [ZCCHC9](http://www.ncbi.nlm.nih.gov/sites/entrez?Db=gene&Cmd=ShowDetailView&TermToSearch=84240) | zinc finger, CCHC domain containing 9 |
| [STX16](http://www.ncbi.nlm.nih.gov/sites/entrez?Db=gene&Cmd=ShowDetailView&TermToSearch=8675) | syntaxin 16 |
| [GABRA1](http://www.ncbi.nlm.nih.gov/sites/entrez?Db=gene&Cmd=ShowDetailView&TermToSearch=2554) | gamma-aminobutyric acid (GABA) A receptor, alpha 1 |
| [PDIK1L](http://www.ncbi.nlm.nih.gov/sites/entrez?Db=gene&Cmd=ShowDetailView&TermToSearch=149420) | PDLIM1 interacting kinase 1 like |
| [BCLAF1](http://www.ncbi.nlm.nih.gov/sites/entrez?Db=gene&Cmd=ShowDetailView&TermToSearch=9774) | BCL2-associated transcription factor 1 |
| [C1orf83](http://www.ncbi.nlm.nih.gov/sites/entrez?Db=gene&Cmd=ShowDetailView&TermToSearch=127428) | chromosome 1 open reading frame 83 |
| [HEXIM1](http://www.ncbi.nlm.nih.gov/sites/entrez?Db=gene&Cmd=ShowDetailView&TermToSearch=10614) | hexamethylene bis-acetamide inducible 1 |
| [XBP1](http://www.ncbi.nlm.nih.gov/sites/entrez?Db=gene&Cmd=ShowDetailView&TermToSearch=7494) | X-box binding protein 1 |
| [IPO5](http://www.ncbi.nlm.nih.gov/sites/entrez?Db=gene&Cmd=ShowDetailView&TermToSearch=3843) | importin 5 |
| [XPO4](http://www.ncbi.nlm.nih.gov/sites/entrez?Db=gene&Cmd=ShowDetailView&TermToSearch=64328) | exportin 4 |
| [ZNF295](http://www.ncbi.nlm.nih.gov/sites/entrez?Db=gene&Cmd=ShowDetailView&TermToSearch=49854) | zinc finger protein 295 |
| [OPCML](http://www.ncbi.nlm.nih.gov/sites/entrez?Db=gene&Cmd=ShowDetailView&TermToSearch=4978) | opioid binding protein/cell adhesion molecule-like |
| [PKP2](http://www.ncbi.nlm.nih.gov/sites/entrez?Db=gene&Cmd=ShowDetailView&TermToSearch=5318) | plakophilin 2 |
| [ABCB10](http://www.ncbi.nlm.nih.gov/sites/entrez?Db=gene&Cmd=ShowDetailView&TermToSearch=23456) | ATP-binding cassette, sub-family B (MDR/TAP), member 10 |
| [TOP2B](http://www.ncbi.nlm.nih.gov/sites/entrez?Db=gene&Cmd=ShowDetailView&TermToSearch=7155) | topoisomerase (DNA) II beta 180kDa |
| [TMEM38B](http://www.ncbi.nlm.nih.gov/sites/entrez?Db=gene&Cmd=ShowDetailView&TermToSearch=55151) | transmembrane protein 38B |
| [ELF2](http://www.ncbi.nlm.nih.gov/sites/entrez?Db=gene&Cmd=ShowDetailView&TermToSearch=1998) | E74-like factor 2 (ets domain transcription factor) |
| [KCNK2](http://www.ncbi.nlm.nih.gov/sites/entrez?Db=gene&Cmd=ShowDetailView&TermToSearch=3776) | potassium channel, subfamily K, member 2 |
| [ZFP14](http://www.ncbi.nlm.nih.gov/sites/entrez?Db=gene&Cmd=ShowDetailView&TermToSearch=57677) | zinc finger protein 14 homolog (mouse) |
| [FAM20B](http://www.ncbi.nlm.nih.gov/sites/entrez?Db=gene&Cmd=ShowDetailView&TermToSearch=9917) | family with sequence similarity 20, member B |
| [BOLL](http://www.ncbi.nlm.nih.gov/sites/entrez?Db=gene&Cmd=ShowDetailView&TermToSearch=66037) | bol, boule-like (Drosophila) |
| [MYCN](http://www.ncbi.nlm.nih.gov/sites/entrez?Db=gene&Cmd=ShowDetailView&TermToSearch=4613) | v-myc myelocytomatosis viral related oncogene, neuroblastoma derived (avian) |
| [MEGF9](http://www.ncbi.nlm.nih.gov/sites/entrez?Db=gene&Cmd=ShowDetailView&TermToSearch=1955) | multiple EGF-like-domains 9 |
| [ESCO1](http://www.ncbi.nlm.nih.gov/sites/entrez?Db=gene&Cmd=ShowDetailView&TermToSearch=114799) | establishment of cohesion 1 homolog 1 (S. cerevisiae) |
| [LRP11](http://www.ncbi.nlm.nih.gov/sites/entrez?Db=gene&Cmd=ShowDetailView&TermToSearch=84918) | low density lipoprotein receptor-related protein 11 |
| [SMC5](http://www.ncbi.nlm.nih.gov/sites/entrez?Db=gene&Cmd=ShowDetailView&TermToSearch=23137) | structural maintenance of chromosomes 5 |
| [STYX](http://www.ncbi.nlm.nih.gov/sites/entrez?Db=gene&Cmd=ShowDetailView&TermToSearch=6815) | serine/threonine/tyrosine interacting protein |
| [COL6A3](http://www.ncbi.nlm.nih.gov/sites/entrez?Db=gene&Cmd=ShowDetailView&TermToSearch=1293) | collagen, type VI, alpha 3 |
| [BCL11B](http://www.ncbi.nlm.nih.gov/sites/entrez?Db=gene&Cmd=ShowDetailView&TermToSearch=64919) | B-cell CLL/lymphoma 11B (zinc finger protein) |
| [SGIP1](http://www.ncbi.nlm.nih.gov/sites/entrez?Db=gene&Cmd=ShowDetailView&TermToSearch=84251) | SH3-domain GRB2-like (endophilin) interacting protein 1 |
| [TMEM65](http://www.ncbi.nlm.nih.gov/sites/entrez?Db=gene&Cmd=ShowDetailView&TermToSearch=157378) | transmembrane protein 65 |
| [KIAA1486](http://www.ncbi.nlm.nih.gov/sites/entrez?Db=gene&Cmd=ShowDetailView&TermToSearch=57624) | KIAA1486 protein |
| [ADRBK2](http://www.ncbi.nlm.nih.gov/sites/entrez?Db=gene&Cmd=ShowDetailView&TermToSearch=157) | adrenergic, beta, receptor kinase 2 |
| [HOXA1](http://www.ncbi.nlm.nih.gov/sites/entrez?Db=gene&Cmd=ShowDetailView&TermToSearch=3198) | homeobox A1 |
| [CBL](http://www.ncbi.nlm.nih.gov/sites/entrez?Db=gene&Cmd=ShowDetailView&TermToSearch=867) | Cas-Br-M (murine) ecotropic retroviral transforming sequence |
| [BTG1](http://www.ncbi.nlm.nih.gov/sites/entrez?Db=gene&Cmd=ShowDetailView&TermToSearch=694) | B-cell translocation gene 1, anti-proliferative |
| [CCDC55](http://www.ncbi.nlm.nih.gov/sites/entrez?Db=gene&Cmd=ShowDetailView&TermToSearch=84081) | coiled-coil domain containing 55 |
| [DENR](http://www.ncbi.nlm.nih.gov/sites/entrez?Db=gene&Cmd=ShowDetailView&TermToSearch=8562) | density-regulated protein |
| [DPP10](http://www.ncbi.nlm.nih.gov/sites/entrez?Db=gene&Cmd=ShowDetailView&TermToSearch=57628) | dipeptidyl-peptidase 10 |
| [CCDC34](http://www.ncbi.nlm.nih.gov/sites/entrez?Db=gene&Cmd=ShowDetailView&TermToSearch=91057) | coiled-coil domain containing 34 |
| [PABPC3](http://www.ncbi.nlm.nih.gov/sites/entrez?Db=gene&Cmd=ShowDetailView&TermToSearch=5042) | poly(A) binding protein, cytoplasmic 3 |
| [RAB6B](http://www.ncbi.nlm.nih.gov/sites/entrez?Db=gene&Cmd=ShowDetailView&TermToSearch=51560) | RAB6B, member RAS oncogene family |
| [EIF4A2](http://www.ncbi.nlm.nih.gov/sites/entrez?Db=gene&Cmd=ShowDetailView&TermToSearch=1974) | eukaryotic translation initiation factor 4A, isoform 2 |
| [CALCR](http://www.ncbi.nlm.nih.gov/sites/entrez?Db=gene&Cmd=ShowDetailView&TermToSearch=799) | calcitonin receptor |
| [LHFPL3](http://www.ncbi.nlm.nih.gov/sites/entrez?Db=gene&Cmd=ShowDetailView&TermToSearch=375612) | lipoma HMGIC fusion partner-like 3 |
| [XPA](http://www.ncbi.nlm.nih.gov/sites/entrez?Db=gene&Cmd=ShowDetailView&TermToSearch=7507) | xeroderma pigmentosum, complementation group A |
| [WASL](http://www.ncbi.nlm.nih.gov/sites/entrez?Db=gene&Cmd=ShowDetailView&TermToSearch=8976) | Wiskott-Aldrich syndrome-like |
| [TGDS](http://www.ncbi.nlm.nih.gov/sites/entrez?Db=gene&Cmd=ShowDetailView&TermToSearch=23483) | TDP-glucose 4,6-dehydratase |
| [FUT4](http://www.ncbi.nlm.nih.gov/sites/entrez?Db=gene&Cmd=ShowDetailView&TermToSearch=2526) | fucosyltransferase 4 (alpha (1,3) fucosyltransferase, myeloid-specific) |
| [PDE8A](http://www.ncbi.nlm.nih.gov/sites/entrez?Db=gene&Cmd=ShowDetailView&TermToSearch=5151) | phosphodiesterase 8A |
| [PPP6C](http://www.ncbi.nlm.nih.gov/sites/entrez?Db=gene&Cmd=ShowDetailView&TermToSearch=5537) | protein phosphatase 6, catalytic subunit |
| [ZNF831](http://www.ncbi.nlm.nih.gov/sites/entrez?Db=gene&Cmd=ShowDetailView&TermToSearch=128611) | zinc finger protein 831 |
| [MYST3](http://www.ncbi.nlm.nih.gov/sites/entrez?Db=gene&Cmd=ShowDetailView&TermToSearch=7994) | MYST histone acetyltransferase (monocytic leukemia) 3 |
| [MSX1](http://www.ncbi.nlm.nih.gov/sites/entrez?Db=gene&Cmd=ShowDetailView&TermToSearch=4487) | msh homeobox 1 |
| [EML1](http://www.ncbi.nlm.nih.gov/sites/entrez?Db=gene&Cmd=ShowDetailView&TermToSearch=2009) | echinoderm microtubule associated protein like 1 |
| [KRT12](http://www.ncbi.nlm.nih.gov/sites/entrez?Db=gene&Cmd=ShowDetailView&TermToSearch=3859) | keratin 12 (Meesmann corneal dystrophy) |
| [LUZP4](http://www.ncbi.nlm.nih.gov/sites/entrez?Db=gene&Cmd=ShowDetailView&TermToSearch=51213) | leucine zipper protein 4 |
| [FLJ30851](http://www.ncbi.nlm.nih.gov/sites/entrez?Db=gene&Cmd=ShowDetailView&TermToSearch=653140) | hypothetical LOC653140 |
| [EDEM1](http://www.ncbi.nlm.nih.gov/sites/entrez?Db=gene&Cmd=ShowDetailView&TermToSearch=9695) | ER degradation enhancer, mannosidase alpha-like 1 |
| [AGL](http://www.ncbi.nlm.nih.gov/sites/entrez?Db=gene&Cmd=ShowDetailView&TermToSearch=178) | amylo-1, 6-glucosidase, 4-alpha-glucanotransferase (glycogen debranching enzyme, glycogen storage disease type III) |
| [ZFHX4](http://www.ncbi.nlm.nih.gov/sites/entrez?Db=gene&Cmd=ShowDetailView&TermToSearch=79776) | zinc finger homeobox 4 |
| [SMG1](http://www.ncbi.nlm.nih.gov/sites/entrez?Db=gene&Cmd=ShowDetailView&TermToSearch=23049) | PI-3-kinase-related kinase SMG-1 |
| [PEX5](http://www.ncbi.nlm.nih.gov/sites/entrez?Db=gene&Cmd=ShowDetailView&TermToSearch=5830) | peroxisomal biogenesis factor 5 |
| [GABRB3](http://www.ncbi.nlm.nih.gov/sites/entrez?Db=gene&Cmd=ShowDetailView&TermToSearch=2562) | gamma-aminobutyric acid (GABA) A receptor, beta 3 |
| [NOL1](http://www.ncbi.nlm.nih.gov/sites/entrez?Db=gene&Cmd=ShowDetailView&TermToSearch=4839) | nucleolar protein 1, 120kDa |
| [TRIP12](http://www.ncbi.nlm.nih.gov/sites/entrez?Db=gene&Cmd=ShowDetailView&TermToSearch=9320) | thyroid hormone receptor interactor 12 |
| [CHODL](http://www.ncbi.nlm.nih.gov/sites/entrez?Db=gene&Cmd=ShowDetailView&TermToSearch=140578) | chondrolectin |
| [PCSK2](http://www.ncbi.nlm.nih.gov/sites/entrez?Db=gene&Cmd=ShowDetailView&TermToSearch=5126) | proprotein convertase subtilisin/kexin type 2 |
| [HDX](http://www.ncbi.nlm.nih.gov/sites/entrez?Db=gene&Cmd=ShowDetailView&TermToSearch=139324) | highly divergent homeobox |
| [KLF3](http://www.ncbi.nlm.nih.gov/sites/entrez?Db=gene&Cmd=ShowDetailView&TermToSearch=51274) | Kruppel-like factor 3 (basic) |
| [C7orf60](http://www.ncbi.nlm.nih.gov/sites/entrez?Db=gene&Cmd=ShowDetailView&TermToSearch=154743) | chromosome 7 open reading frame 60 |
| [ARHGEF9](http://www.ncbi.nlm.nih.gov/sites/entrez?Db=gene&Cmd=ShowDetailView&TermToSearch=23229) | Cdc42 guanine nucleotide exchange factor (GEF) 9 |
| [TANC1](http://www.ncbi.nlm.nih.gov/sites/entrez?Db=gene&Cmd=ShowDetailView&TermToSearch=85461) | tetratricopeptide repeat, ankyrin repeat and coiled-coil containing 1 |
| [SMEK1](http://www.ncbi.nlm.nih.gov/sites/entrez?Db=gene&Cmd=ShowDetailView&TermToSearch=55671) | SMEK homolog 1, suppressor of mek1 (Dictyostelium) |
| [PPP3CA](http://www.ncbi.nlm.nih.gov/sites/entrez?Db=gene&Cmd=ShowDetailView&TermToSearch=5530) | protein phosphatase 3 (formerly 2B), catalytic subunit, alpha isoform |
| [PBX2](http://www.ncbi.nlm.nih.gov/sites/entrez?Db=gene&Cmd=ShowDetailView&TermToSearch=5089) | pre-B-cell leukemia homeobox 2 |
| [SOX30](http://www.ncbi.nlm.nih.gov/sites/entrez?Db=gene&Cmd=ShowDetailView&TermToSearch=11063) | SRY (sex determining region Y)-box 30 |
| [SF1](http://www.ncbi.nlm.nih.gov/sites/entrez?Db=gene&Cmd=ShowDetailView&TermToSearch=7536) | splicing factor 1 |
| [UBR7](http://www.ncbi.nlm.nih.gov/sites/entrez?Db=gene&Cmd=ShowDetailView&TermToSearch=55148) | ubiquitin protein ligase E3 component n-recognin 7 (putative) |
| [NOVA1](http://www.ncbi.nlm.nih.gov/sites/entrez?Db=gene&Cmd=ShowDetailView&TermToSearch=4857) | neuro-oncological ventral antigen 1 |
| [C4orf34](http://www.ncbi.nlm.nih.gov/sites/entrez?Db=gene&Cmd=ShowDetailView&TermToSearch=201895) | chromosome 4 open reading frame 34 |
| [CD55](http://www.ncbi.nlm.nih.gov/sites/entrez?Db=gene&Cmd=ShowDetailView&TermToSearch=1604) | CD55 molecule, decay accelerating factor for complement (Cromer blood group) |
| [RAP2C](http://www.ncbi.nlm.nih.gov/sites/entrez?Db=gene&Cmd=ShowDetailView&TermToSearch=57826) | RAP2C, member of RAS oncogene family |
| [EFCAB5](http://www.ncbi.nlm.nih.gov/sites/entrez?Db=gene&Cmd=ShowDetailView&TermToSearch=374786) | EF-hand calcium binding domain 5 |
| [ZNF618](http://www.ncbi.nlm.nih.gov/sites/entrez?Db=gene&Cmd=ShowDetailView&TermToSearch=114991) | zinc finger protein 618 |
| [MTF1](http://www.ncbi.nlm.nih.gov/sites/entrez?Db=gene&Cmd=ShowDetailView&TermToSearch=4520) | metal-regulatory transcription factor 1 |
| [MKRN2](http://www.ncbi.nlm.nih.gov/sites/entrez?Db=gene&Cmd=ShowDetailView&TermToSearch=23609) | makorin, ring finger protein, 2 |
| [TXNDC5](http://www.ncbi.nlm.nih.gov/sites/entrez?Db=gene&Cmd=ShowDetailView&TermToSearch=81567) | thioredoxin domain containing 5 |
| [CNTNAP2](http://www.ncbi.nlm.nih.gov/sites/entrez?Db=gene&Cmd=ShowDetailView&TermToSearch=26047) | contactin associated protein-like 2 |
| [RWDD4A](http://www.ncbi.nlm.nih.gov/sites/entrez?Db=gene&Cmd=ShowDetailView&TermToSearch=201965) | RWD domain containing 4A |
| [tcag7.1228](http://www.ncbi.nlm.nih.gov/sites/entrez?Db=gene&Cmd=ShowDetailView&TermToSearch=254048) | hypothetical protein FLJ25778 |
| [YTHDC1](http://www.ncbi.nlm.nih.gov/sites/entrez?Db=gene&Cmd=ShowDetailView&TermToSearch=91746) | YTH domain containing 1 |
| [SMAD4](http://www.ncbi.nlm.nih.gov/sites/entrez?Db=gene&Cmd=ShowDetailView&TermToSearch=4089) | SMAD family member 4 |
| [KCNIP4](http://www.ncbi.nlm.nih.gov/sites/entrez?Db=gene&Cmd=ShowDetailView&TermToSearch=80333) | Kv channel interacting protein 4 |
| [SYNGAP1](http://www.ncbi.nlm.nih.gov/sites/entrez?Db=gene&Cmd=ShowDetailView&TermToSearch=8831) | synaptic Ras GTPase activating protein 1 homolog (rat) |
| [NPTX2](http://www.ncbi.nlm.nih.gov/sites/entrez?Db=gene&Cmd=ShowDetailView&TermToSearch=4885) | neuronal pentraxin II |
| [SLC8A1](http://www.ncbi.nlm.nih.gov/sites/entrez?Db=gene&Cmd=ShowDetailView&TermToSearch=6546) | solute carrier family 8 (sodium/calcium exchanger), member 1 |
| [UBE2L3](http://www.ncbi.nlm.nih.gov/sites/entrez?Db=gene&Cmd=ShowDetailView&TermToSearch=7332) | ubiquitin-conjugating enzyme E2L 3 |
| [HIRA](http://www.ncbi.nlm.nih.gov/sites/entrez?Db=gene&Cmd=ShowDetailView&TermToSearch=7290) | HIR histone cell cycle regulation defective homolog A (S. cerevisiae) |
| [TSHZ3](http://www.ncbi.nlm.nih.gov/sites/entrez?Db=gene&Cmd=ShowDetailView&TermToSearch=57616) | teashirt zinc finger homeobox 3 |
| [ZNF335](http://www.ncbi.nlm.nih.gov/sites/entrez?Db=gene&Cmd=ShowDetailView&TermToSearch=63925) | zinc finger protein 335 |
| [PATL1](http://www.ncbi.nlm.nih.gov/sites/entrez?Db=gene&Cmd=ShowDetailView&TermToSearch=219988) | protein associated with topoisomerase II homolog 1 (yeast) |
| [FAM33A](http://www.ncbi.nlm.nih.gov/sites/entrez?Db=gene&Cmd=ShowDetailView&TermToSearch=348235) | family with sequence similarity 33, member A |
| [SUZ12](http://www.ncbi.nlm.nih.gov/sites/entrez?Db=gene&Cmd=ShowDetailView&TermToSearch=23512) | suppressor of zeste 12 homolog (Drosophila) |
| [ELL2](http://www.ncbi.nlm.nih.gov/sites/entrez?Db=gene&Cmd=ShowDetailView&TermToSearch=22936) | elongation factor, RNA polymerase II, 2 |
| [CCDC131](http://www.ncbi.nlm.nih.gov/sites/entrez?Db=gene&Cmd=ShowDetailView&TermToSearch=196441) | coiled-coil domain containing 131 |
| [FZD3](http://www.ncbi.nlm.nih.gov/sites/entrez?Db=gene&Cmd=ShowDetailView&TermToSearch=7976) | frizzled homolog 3 (Drosophila) |
| [ATXN7L1](http://www.ncbi.nlm.nih.gov/sites/entrez?Db=gene&Cmd=ShowDetailView&TermToSearch=222255) | ataxin 7-like 1 |
| [IRS2](http://www.ncbi.nlm.nih.gov/sites/entrez?Db=gene&Cmd=ShowDetailView&TermToSearch=8660) | insulin receptor substrate 2 |
| [FBXL7](http://www.ncbi.nlm.nih.gov/sites/entrez?Db=gene&Cmd=ShowDetailView&TermToSearch=23194) | F-box and leucine-rich repeat protein 7 |
| [SHANK2](http://www.ncbi.nlm.nih.gov/sites/entrez?Db=gene&Cmd=ShowDetailView&TermToSearch=22941) | SH3 and multiple ankyrin repeat domains 2 |
| [ZNF518A](http://www.ncbi.nlm.nih.gov/sites/entrez?Db=gene&Cmd=ShowDetailView&TermToSearch=9849) | zinc finger protein 518A |
| [ZCCHC14](http://www.ncbi.nlm.nih.gov/sites/entrez?Db=gene&Cmd=ShowDetailView&TermToSearch=23174) | zinc finger, CCHC domain containing 14 |
| [RBM4B](http://www.ncbi.nlm.nih.gov/sites/entrez?Db=gene&Cmd=ShowDetailView&TermToSearch=83759) | RNA binding motif protein 4B |
| [KIAA1430](http://www.ncbi.nlm.nih.gov/sites/entrez?Db=gene&Cmd=ShowDetailView&TermToSearch=57587) | KIAA1430 |
| [PDE4D](http://www.ncbi.nlm.nih.gov/sites/entrez?Db=gene&Cmd=ShowDetailView&TermToSearch=5144) | phosphodiesterase 4D, cAMP-specific (phosphodiesterase E3 dunce homolog, Drosophila) |
| [AKAP9](http://www.ncbi.nlm.nih.gov/sites/entrez?Db=gene&Cmd=ShowDetailView&TermToSearch=10142) | A kinase (PRKA) anchor protein (yotiao) 9 |
| [SLC9A2](http://www.ncbi.nlm.nih.gov/sites/entrez?Db=gene&Cmd=ShowDetailView&TermToSearch=6549) | solute carrier family 9 (sodium/hydrogen exchanger), member 2 |
| [DAZL](http://www.ncbi.nlm.nih.gov/sites/entrez?Db=gene&Cmd=ShowDetailView&TermToSearch=1618) | deleted in azoospermia-like |
| [DSC2](http://www.ncbi.nlm.nih.gov/sites/entrez?Db=gene&Cmd=ShowDetailView&TermToSearch=1824) | desmocollin 2 |
| [AHDC1](http://www.ncbi.nlm.nih.gov/sites/entrez?Db=gene&Cmd=ShowDetailView&TermToSearch=27245) | AT hook, DNA binding motif, containing 1 |
| [FAM84A](http://www.ncbi.nlm.nih.gov/sites/entrez?Db=gene&Cmd=ShowDetailView&TermToSearch=151354) | family with sequence similarity 84, member A |
| [SHISA2](http://www.ncbi.nlm.nih.gov/sites/entrez?Db=gene&Cmd=ShowDetailView&TermToSearch=387914) | shisa homolog 2 (Xenopus laevis) |
| [C6orf35](http://www.ncbi.nlm.nih.gov/sites/entrez?Db=gene&Cmd=ShowDetailView&TermToSearch=729515) | chromosome 6 open reading frame 35 |
| [FOXO1](http://www.ncbi.nlm.nih.gov/sites/entrez?Db=gene&Cmd=ShowDetailView&TermToSearch=2308) | forkhead box O1 |
| [REPS2](http://www.ncbi.nlm.nih.gov/sites/entrez?Db=gene&Cmd=ShowDetailView&TermToSearch=9185) | RALBP1 associated Eps domain containing 2 |
| [KLRA1](http://www.ncbi.nlm.nih.gov/sites/entrez?Db=gene&Cmd=ShowDetailView&TermToSearch=10748) | killer cell lectin-like receptor subfamily A, member 1 |
| [PNPLA8](http://www.ncbi.nlm.nih.gov/sites/entrez?Db=gene&Cmd=ShowDetailView&TermToSearch=50640) | patatin-like phospholipase domain containing 8 |
| [SOX5](http://www.ncbi.nlm.nih.gov/sites/entrez?Db=gene&Cmd=ShowDetailView&TermToSearch=6660) | SRY (sex determining region Y)-box 5 |
| [TOR1AIP1](http://www.ncbi.nlm.nih.gov/sites/entrez?Db=gene&Cmd=ShowDetailView&TermToSearch=26092) | torsin A interacting protein 1 |
| [USP22](http://www.ncbi.nlm.nih.gov/sites/entrez?Db=gene&Cmd=ShowDetailView&TermToSearch=23326) | ubiquitin specific peptidase 22 |
| [PDP2](http://www.ncbi.nlm.nih.gov/sites/entrez?Db=gene&Cmd=ShowDetailView&TermToSearch=57546) | pyruvate dehydrogenase phosphatase isoenzyme 2 |
| [SMAD7](http://www.ncbi.nlm.nih.gov/sites/entrez?Db=gene&Cmd=ShowDetailView&TermToSearch=4092) | SMAD family member 7 |
| [SFRS1](http://www.ncbi.nlm.nih.gov/sites/entrez?Db=gene&Cmd=ShowDetailView&TermToSearch=6426) | splicing factor, arginine/serine-rich 1 (splicing factor 2, alternate splicing factor) |
| [RPS6KA3](http://www.ncbi.nlm.nih.gov/sites/entrez?Db=gene&Cmd=ShowDetailView&TermToSearch=6197) | ribosomal protein S6 kinase, 90kDa, polypeptide 3 |
| [PDGFRA](http://www.ncbi.nlm.nih.gov/sites/entrez?Db=gene&Cmd=ShowDetailView&TermToSearch=5156) | platelet-derived growth factor receptor, alpha polypeptide |
| [FBXW7](http://www.ncbi.nlm.nih.gov/sites/entrez?Db=gene&Cmd=ShowDetailView&TermToSearch=55294) | F-box and WD repeat domain containing 7 |
| [SPIN3](http://www.ncbi.nlm.nih.gov/sites/entrez?Db=gene&Cmd=ShowDetailView&TermToSearch=169981) | spindlin family, member 3 |
| [RAB22A](http://www.ncbi.nlm.nih.gov/sites/entrez?Db=gene&Cmd=ShowDetailView&TermToSearch=57403) | RAB22A, member RAS oncogene family |
| [MAFK](http://www.ncbi.nlm.nih.gov/sites/entrez?Db=gene&Cmd=ShowDetailView&TermToSearch=7975) | v-maf musculoaponeurotic fibrosarcoma oncogene homolog K (avian) |
| [RNF44](http://www.ncbi.nlm.nih.gov/sites/entrez?Db=gene&Cmd=ShowDetailView&TermToSearch=22838) | ring finger protein 44 |
| [TET3](http://www.ncbi.nlm.nih.gov/sites/entrez?Db=gene&Cmd=ShowDetailView&TermToSearch=200424) | tet oncogene family member 3 |
| [WNK3](http://www.ncbi.nlm.nih.gov/sites/entrez?Db=gene&Cmd=ShowDetailView&TermToSearch=65267) | WNK lysine deficient protein kinase 3 |
| [P4HB](http://www.ncbi.nlm.nih.gov/sites/entrez?Db=gene&Cmd=ShowDetailView&TermToSearch=5034) | procollagen-proline, 2-oxoglutarate 4-dioxygenase (proline 4-hydroxylase), beta polypeptide |
| [EDA](http://www.ncbi.nlm.nih.gov/sites/entrez?Db=gene&Cmd=ShowDetailView&TermToSearch=1896) | ectodysplasin A |
| [TNFSF15](http://www.ncbi.nlm.nih.gov/sites/entrez?Db=gene&Cmd=ShowDetailView&TermToSearch=9966) | tumor necrosis factor (ligand) superfamily, member 15 |
| [KCMF1](http://www.ncbi.nlm.nih.gov/sites/entrez?Db=gene&Cmd=ShowDetailView&TermToSearch=56888) | potassium channel modulatory factor 1 |
| [TNFAIP3](http://www.ncbi.nlm.nih.gov/sites/entrez?Db=gene&Cmd=ShowDetailView&TermToSearch=7128) | tumor necrosis factor, alpha-induced protein 3 |
| [SLC6A9](http://www.ncbi.nlm.nih.gov/sites/entrez?Db=gene&Cmd=ShowDetailView&TermToSearch=6536) | solute carrier family 6 (neurotransmitter transporter, glycine), member 9 |
| [ZNF516](http://www.ncbi.nlm.nih.gov/sites/entrez?Db=gene&Cmd=ShowDetailView&TermToSearch=9658) | zinc finger protein 516 |
| [NUP153](http://www.ncbi.nlm.nih.gov/sites/entrez?Db=gene&Cmd=ShowDetailView&TermToSearch=9972) | nucleoporin 153kDa |
| [HP1BP3](http://www.ncbi.nlm.nih.gov/sites/entrez?Db=gene&Cmd=ShowDetailView&TermToSearch=50809) | heterochromatin protein 1, binding protein 3 |
| [HIPK2](http://www.ncbi.nlm.nih.gov/sites/entrez?Db=gene&Cmd=ShowDetailView&TermToSearch=28996) | homeodomain interacting protein kinase 2 |
| [BRD4](http://www.ncbi.nlm.nih.gov/sites/entrez?Db=gene&Cmd=ShowDetailView&TermToSearch=23476) | bromodomain containing 4 |
| [GPR85](http://www.ncbi.nlm.nih.gov/sites/entrez?Db=gene&Cmd=ShowDetailView&TermToSearch=54329) | G protein-coupled receptor 85 |
| [CPNE4](http://www.ncbi.nlm.nih.gov/sites/entrez?Db=gene&Cmd=ShowDetailView&TermToSearch=131034) | copine IV |
| [IQCK](http://www.ncbi.nlm.nih.gov/sites/entrez?Db=gene&Cmd=ShowDetailView&TermToSearch=124152) | IQ motif containing K |
| [PAFAH1B1](http://www.ncbi.nlm.nih.gov/sites/entrez?Db=gene&Cmd=ShowDetailView&TermToSearch=5048) | platelet-activating factor acetylhydrolase, isoform Ib, alpha subunit 45kDa |
| [CCDC24](http://www.ncbi.nlm.nih.gov/sites/entrez?Db=gene&Cmd=ShowDetailView&TermToSearch=149473) | coiled-coil domain containing 24 |
| [AMTN](http://www.ncbi.nlm.nih.gov/sites/entrez?Db=gene&Cmd=ShowDetailView&TermToSearch=401138) | amelotin |
| [ATP11C](http://www.ncbi.nlm.nih.gov/sites/entrez?Db=gene&Cmd=ShowDetailView&TermToSearch=286410) | ATPase, class VI, type 11C |
| [TSC1](http://www.ncbi.nlm.nih.gov/sites/entrez?Db=gene&Cmd=ShowDetailView&TermToSearch=7248) | tuberous sclerosis 1 |
| [SLC35A5](http://www.ncbi.nlm.nih.gov/sites/entrez?Db=gene&Cmd=ShowDetailView&TermToSearch=55032) | solute carrier family 35, member A5 |
| [KIAA1244](http://www.ncbi.nlm.nih.gov/sites/entrez?Db=gene&Cmd=ShowDetailView&TermToSearch=57221) | KIAA1244 |
| [EWSR1](http://www.ncbi.nlm.nih.gov/sites/entrez?Db=gene&Cmd=ShowDetailView&TermToSearch=2130) | Ewing sarcoma breakpoint region 1 |
| [TPPP](http://www.ncbi.nlm.nih.gov/sites/entrez?Db=gene&Cmd=ShowDetailView&TermToSearch=11076) | tubulin polymerization promoting protein |
| [THRB](http://www.ncbi.nlm.nih.gov/sites/entrez?Db=gene&Cmd=ShowDetailView&TermToSearch=7068) | thyroid hormone receptor, beta (erythroblastic leukemia viral (v-erb-a) oncogene homolog 2, avian) |
| [DYRK2](http://www.ncbi.nlm.nih.gov/sites/entrez?Db=gene&Cmd=ShowDetailView&TermToSearch=8445) | dual-specificity tyrosine-(Y)-phosphorylation regulated kinase 2 |
| [XPO5](http://www.ncbi.nlm.nih.gov/sites/entrez?Db=gene&Cmd=ShowDetailView&TermToSearch=57510) | exportin 5 |
| [BCL11A](http://www.ncbi.nlm.nih.gov/sites/entrez?Db=gene&Cmd=ShowDetailView&TermToSearch=53335) | B-cell CLL/lymphoma 11A (zinc finger protein) |
| [MGAT4A](http://www.ncbi.nlm.nih.gov/sites/entrez?Db=gene&Cmd=ShowDetailView&TermToSearch=11320) | mannosyl (alpha-1,3-)-glycoprotein beta-1,4-N-acetylglucosaminyltransferase, isozyme A |
| [NOLC1](http://www.ncbi.nlm.nih.gov/sites/entrez?Db=gene&Cmd=ShowDetailView&TermToSearch=9221) | nucleolar and coiled-body phosphoprotein 1 |
| [LRIG1](http://www.ncbi.nlm.nih.gov/sites/entrez?Db=gene&Cmd=ShowDetailView&TermToSearch=26018) | leucine-rich repeats and immunoglobulin-like domains 1 |
| [NRF1](http://www.ncbi.nlm.nih.gov/sites/entrez?Db=gene&Cmd=ShowDetailView&TermToSearch=4899) | nuclear respiratory factor 1 |
| [FERMT1](http://www.ncbi.nlm.nih.gov/sites/entrez?Db=gene&Cmd=ShowDetailView&TermToSearch=55612) | fermitin family homolog 1 (Drosophila) |
| [RNF122](http://www.ncbi.nlm.nih.gov/sites/entrez?Db=gene&Cmd=ShowDetailView&TermToSearch=79845) | ring finger protein 122 |
| [STX1A](http://www.ncbi.nlm.nih.gov/sites/entrez?Db=gene&Cmd=ShowDetailView&TermToSearch=6804) | syntaxin 1A (brain) |
| [C5orf41](http://www.ncbi.nlm.nih.gov/sites/entrez?Db=gene&Cmd=ShowDetailView&TermToSearch=153222) | chromosome 5 open reading frame 41 |
| [TBX3](http://www.ncbi.nlm.nih.gov/sites/entrez?Db=gene&Cmd=ShowDetailView&TermToSearch=6926) | T-box 3 (ulnar mammary syndrome) |
| [TJAP1](http://www.ncbi.nlm.nih.gov/sites/entrez?Db=gene&Cmd=ShowDetailView&TermToSearch=93643) | tight junction associated protein 1 (peripheral) |
| [XPO1](http://www.ncbi.nlm.nih.gov/sites/entrez?Db=gene&Cmd=ShowDetailView&TermToSearch=7514) | exportin 1 (CRM1 homolog, yeast) |
| [TMEM184A](http://www.ncbi.nlm.nih.gov/sites/entrez?Db=gene&Cmd=ShowDetailView&TermToSearch=202915) | transmembrane protein 184A |
| [WWC3](http://www.ncbi.nlm.nih.gov/sites/entrez?Db=gene&Cmd=ShowDetailView&TermToSearch=55841) | WWC family member 3 |
| [LRRC4](http://www.ncbi.nlm.nih.gov/sites/entrez?Db=gene&Cmd=ShowDetailView&TermToSearch=64101) | leucine rich repeat containing 4 |
| [IER5L](http://www.ncbi.nlm.nih.gov/sites/entrez?Db=gene&Cmd=ShowDetailView&TermToSearch=389792) | immediate early response 5-like |
| [PTPRZ1](http://www.ncbi.nlm.nih.gov/sites/entrez?Db=gene&Cmd=ShowDetailView&TermToSearch=5803) | protein tyrosine phosphatase, receptor-type, Z polypeptide 1 |
| [ZAK](http://www.ncbi.nlm.nih.gov/sites/entrez?Db=gene&Cmd=ShowDetailView&TermToSearch=51776) | sterile alpha motif and leucine zipper containing kinase AZK |
| [EFNB3](http://www.ncbi.nlm.nih.gov/sites/entrez?Db=gene&Cmd=ShowDetailView&TermToSearch=1949) | ephrin-B3 |
| [MEIS1](http://www.ncbi.nlm.nih.gov/sites/entrez?Db=gene&Cmd=ShowDetailView&TermToSearch=4211) | Meis homeobox 1 |
| [MPHOSPH8](http://www.ncbi.nlm.nih.gov/sites/entrez?Db=gene&Cmd=ShowDetailView&TermToSearch=54737) | M-phase phosphoprotein 8 |
| [WDHD1](http://www.ncbi.nlm.nih.gov/sites/entrez?Db=gene&Cmd=ShowDetailView&TermToSearch=11169) | WD repeat and HMG-box DNA binding protein 1 |
| [PURB](http://www.ncbi.nlm.nih.gov/sites/entrez?Db=gene&Cmd=ShowDetailView&TermToSearch=5814) | purine-rich element binding protein B |
| [PAPD5](http://www.ncbi.nlm.nih.gov/sites/entrez?Db=gene&Cmd=ShowDetailView&TermToSearch=64282) | PAP associated domain containing 5 |
| [NOX1](http://www.ncbi.nlm.nih.gov/sites/entrez?Db=gene&Cmd=ShowDetailView&TermToSearch=27035) | NADPH oxidase 1 |
| [MTMR12](http://www.ncbi.nlm.nih.gov/sites/entrez?Db=gene&Cmd=ShowDetailView&TermToSearch=54545) | myotubularin related protein 12 |
| [VASP](http://www.ncbi.nlm.nih.gov/sites/entrez?Db=gene&Cmd=ShowDetailView&TermToSearch=7408) | vasodilator-stimulated phosphoprotein |
| [IGF1](http://www.ncbi.nlm.nih.gov/sites/entrez?Db=gene&Cmd=ShowDetailView&TermToSearch=3479) | insulin-like growth factor 1 (somatomedin C) |
| [MYCBP2](http://www.ncbi.nlm.nih.gov/sites/entrez?Db=gene&Cmd=ShowDetailView&TermToSearch=23077) | MYC binding protein 2 |
| [TNRC6B](http://www.ncbi.nlm.nih.gov/sites/entrez?Db=gene&Cmd=ShowDetailView&TermToSearch=23112) | trinucleotide repeat containing 6B |
| [QSOX2](http://www.ncbi.nlm.nih.gov/sites/entrez?Db=gene&Cmd=ShowDetailView&TermToSearch=169714) | quiescin Q6 sulfhydryl oxidase 2 |
| [MYCL1](http://www.ncbi.nlm.nih.gov/sites/entrez?Db=gene&Cmd=ShowDetailView&TermToSearch=4610) | v-myc myelocytomatosis viral oncogene homolog 1, lung carcinoma derived (avian) |
| [VAPB](http://www.ncbi.nlm.nih.gov/sites/entrez?Db=gene&Cmd=ShowDetailView&TermToSearch=9217) | VAMP (vesicle-associated membrane protein)-associated protein B and C |
| [FAM81A](http://www.ncbi.nlm.nih.gov/sites/entrez?Db=gene&Cmd=ShowDetailView&TermToSearch=145773) | family with sequence similarity 81, member A |
| [MOBKL2B](http://www.ncbi.nlm.nih.gov/sites/entrez?Db=gene&Cmd=ShowDetailView&TermToSearch=79817) | MOB1, Mps One Binder kinase activator-like 2B (yeast) |
| [MSC](http://www.ncbi.nlm.nih.gov/sites/entrez?Db=gene&Cmd=ShowDetailView&TermToSearch=9242) | musculin (activated B-cell factor-1) |
| [USP34](http://www.ncbi.nlm.nih.gov/sites/entrez?Db=gene&Cmd=ShowDetailView&TermToSearch=9736) | ubiquitin specific peptidase 34 |
| [FGF9](http://www.ncbi.nlm.nih.gov/sites/entrez?Db=gene&Cmd=ShowDetailView&TermToSearch=2254) | fibroblast growth factor 9 (glia-activating factor) |
| [SOBP](http://www.ncbi.nlm.nih.gov/sites/entrez?Db=gene&Cmd=ShowDetailView&TermToSearch=55084) | sine oculis binding protein homolog (Drosophila) |
| [CITED2](http://www.ncbi.nlm.nih.gov/sites/entrez?Db=gene&Cmd=ShowDetailView&TermToSearch=10370) | Cbp/p300-interacting transactivator, with Glu/Asp-rich carboxy-terminal domain, 2 |
| [SP1](http://www.ncbi.nlm.nih.gov/sites/entrez?Db=gene&Cmd=ShowDetailView&TermToSearch=6667) | Sp1 transcription factor |
| [KRAS](http://www.ncbi.nlm.nih.gov/sites/entrez?Db=gene&Cmd=ShowDetailView&TermToSearch=3845) | v-Ki-ras2 Kirsten rat sarcoma viral oncogene homolog |
| [MECP2](http://www.ncbi.nlm.nih.gov/sites/entrez?Db=gene&Cmd=ShowDetailView&TermToSearch=4204) | methyl CpG binding protein 2 (Rett syndrome) |
| [GLUD1](http://www.ncbi.nlm.nih.gov/sites/entrez?Db=gene&Cmd=ShowDetailView&TermToSearch=2746) | glutamate dehydrogenase 1 |
| [FSTL4](http://www.ncbi.nlm.nih.gov/sites/entrez?Db=gene&Cmd=ShowDetailView&TermToSearch=23105) | follistatin-like 4 |
| [NFIX](http://www.ncbi.nlm.nih.gov/sites/entrez?Db=gene&Cmd=ShowDetailView&TermToSearch=4784) | nuclear factor I/X (CCAAT-binding transcription factor) |
| [SPCS2](http://www.ncbi.nlm.nih.gov/sites/entrez?Db=gene&Cmd=ShowDetailView&TermToSearch=9789) | signal peptidase complex subunit 2 homolog (S. cerevisiae) |
| [C8orf44](http://www.ncbi.nlm.nih.gov/sites/entrez?Db=gene&Cmd=ShowDetailView&TermToSearch=56260) | chromosome 8 open reading frame 44 |
| [SULT4A1](http://www.ncbi.nlm.nih.gov/sites/entrez?Db=gene&Cmd=ShowDetailView&TermToSearch=25830) | sulfotransferase family 4A, member 1 |
| [EIF4G1](http://www.ncbi.nlm.nih.gov/sites/entrez?Db=gene&Cmd=ShowDetailView&TermToSearch=1981) | eukaryotic translation initiation factor 4 gamma, 1 |
| [MYH10](http://www.ncbi.nlm.nih.gov/sites/entrez?Db=gene&Cmd=ShowDetailView&TermToSearch=4628) | myosin, heavy chain 10, non-muscle |
| [VGLL4](http://www.ncbi.nlm.nih.gov/sites/entrez?Db=gene&Cmd=ShowDetailView&TermToSearch=9686) | vestigial like 4 (Drosophila) |
| [RFC1](http://www.ncbi.nlm.nih.gov/sites/entrez?Db=gene&Cmd=ShowDetailView&TermToSearch=5981) | replication factor C (activator 1) 1, 145kDa |
| [BAZ2A](http://www.ncbi.nlm.nih.gov/sites/entrez?Db=gene&Cmd=ShowDetailView&TermToSearch=11176) | bromodomain adjacent to zinc finger domain, 2A |
| [NUDT12](http://www.ncbi.nlm.nih.gov/sites/entrez?Db=gene&Cmd=ShowDetailView&TermToSearch=83594) | nudix (nucleoside diphosphate linked moiety X)-type motif 12 |
| [KLF13](http://www.ncbi.nlm.nih.gov/sites/entrez?Db=gene&Cmd=ShowDetailView&TermToSearch=51621) | Kruppel-like factor 13 |
| [EIF2AK4](http://www.ncbi.nlm.nih.gov/sites/entrez?Db=gene&Cmd=ShowDetailView&TermToSearch=440275) | eukaryotic translation initiation factor 2 alpha kinase 4 |
| [CACNA1C](http://www.ncbi.nlm.nih.gov/sites/entrez?Db=gene&Cmd=ShowDetailView&TermToSearch=775) | calcium channel, voltage-dependent, L type, alpha 1C subunit |
| [UBR1](http://www.ncbi.nlm.nih.gov/sites/entrez?Db=gene&Cmd=ShowDetailView&TermToSearch=197131) | ubiquitin protein ligase E3 component n-recognin 1 |
| [GRIK2](http://www.ncbi.nlm.nih.gov/sites/entrez?Db=gene&Cmd=ShowDetailView&TermToSearch=2898) | glutamate receptor, ionotropic, kainate 2 |
| [ACADSB](http://www.ncbi.nlm.nih.gov/sites/entrez?Db=gene&Cmd=ShowDetailView&TermToSearch=36) | acyl-Coenzyme A dehydrogenase, short/branched chain |
| [SRRM1](http://www.ncbi.nlm.nih.gov/sites/entrez?Db=gene&Cmd=ShowDetailView&TermToSearch=10250) | serine/arginine repetitive matrix 1 |
| [RGS2](http://www.ncbi.nlm.nih.gov/sites/entrez?Db=gene&Cmd=ShowDetailView&TermToSearch=5997) | regulator of G-protein signaling 2, 24kDa |
| [DOCK7](http://www.ncbi.nlm.nih.gov/sites/entrez?Db=gene&Cmd=ShowDetailView&TermToSearch=85440) | dedicator of cytokinesis 7 |
| [STAT3](http://www.ncbi.nlm.nih.gov/sites/entrez?Db=gene&Cmd=ShowDetailView&TermToSearch=6774) | signal transducer and activator of transcription 3 (acute-phase response factor) |
| [C10orf140](http://www.ncbi.nlm.nih.gov/sites/entrez?Db=gene&Cmd=ShowDetailView&TermToSearch=387640) | chromosome 10 open reading frame 140 |
| [SLC20A2](http://www.ncbi.nlm.nih.gov/sites/entrez?Db=gene&Cmd=ShowDetailView&TermToSearch=6575) | solute carrier family 20 (phosphate transporter), member 2 |
| [OSBPL9](http://www.ncbi.nlm.nih.gov/sites/entrez?Db=gene&Cmd=ShowDetailView&TermToSearch=114883) | oxysterol binding protein-like 9 |
| [TRA2A](http://www.ncbi.nlm.nih.gov/sites/entrez?Db=gene&Cmd=ShowDetailView&TermToSearch=29896) | transformer-2 alpha |
| [NEBL](http://www.ncbi.nlm.nih.gov/sites/entrez?Db=gene&Cmd=ShowDetailView&TermToSearch=10529) | nebulette |
| [DLX1](http://www.ncbi.nlm.nih.gov/sites/entrez?Db=gene&Cmd=ShowDetailView&TermToSearch=1745) | distal-less homeobox 1 |
| [DRAP1](http://www.ncbi.nlm.nih.gov/sites/entrez?Db=gene&Cmd=ShowDetailView&TermToSearch=10589) | DR1-associated protein 1 (negative cofactor 2 alpha) |
| [NAB2](http://www.ncbi.nlm.nih.gov/sites/entrez?Db=gene&Cmd=ShowDetailView&TermToSearch=4665) | NGFI-A binding protein 2 (EGR1 binding protein 2) |
| [USP32](http://www.ncbi.nlm.nih.gov/sites/entrez?Db=gene&Cmd=ShowDetailView&TermToSearch=84669) | ubiquitin specific peptidase 32 |
| [RHBDL3](http://www.ncbi.nlm.nih.gov/sites/entrez?Db=gene&Cmd=ShowDetailView&TermToSearch=162494) | rhomboid, veinlet-like 3 (Drosophila) |
| [JMJD1C](http://www.ncbi.nlm.nih.gov/sites/entrez?Db=gene&Cmd=ShowDetailView&TermToSearch=221037) | jumonji domain containing 1C |
| [DACT1](http://www.ncbi.nlm.nih.gov/sites/entrez?Db=gene&Cmd=ShowDetailView&TermToSearch=51339) | dapper, antagonist of beta-catenin, homolog 1 (Xenopus laevis) |
| [POM121C](http://www.ncbi.nlm.nih.gov/sites/entrez?Db=gene&Cmd=ShowDetailView&TermToSearch=100101267) | POM121 membrane glycoprotein C |
| [ANKRD12](http://www.ncbi.nlm.nih.gov/sites/entrez?Db=gene&Cmd=ShowDetailView&TermToSearch=23253) | ankyrin repeat domain 12 |
| [SH3PXD2A](http://www.ncbi.nlm.nih.gov/sites/entrez?Db=gene&Cmd=ShowDetailView&TermToSearch=9644) | SH3 and PX domains 2A |
| [MED15](http://www.ncbi.nlm.nih.gov/sites/entrez?Db=gene&Cmd=ShowDetailView&TermToSearch=51586) | mediator complex subunit 15 |
| [MCOLN2](http://www.ncbi.nlm.nih.gov/sites/entrez?Db=gene&Cmd=ShowDetailView&TermToSearch=255231) | mucolipin 2 |
| [POM121](http://www.ncbi.nlm.nih.gov/sites/entrez?Db=gene&Cmd=ShowDetailView&TermToSearch=9883) | POM121 membrane glycoprotein (rat) |
| [IRF2](http://www.ncbi.nlm.nih.gov/sites/entrez?Db=gene&Cmd=ShowDetailView&TermToSearch=3660) | interferon regulatory factor 2 |
| [KY](http://www.ncbi.nlm.nih.gov/sites/entrez?Db=gene&Cmd=ShowDetailView&TermToSearch=339855) | kyphoscoliosis peptidase |
| [ASCL1](http://www.ncbi.nlm.nih.gov/sites/entrez?Db=gene&Cmd=ShowDetailView&TermToSearch=429) | achaete-scute complex homolog 1 (Drosophila) |
| [DCX](http://www.ncbi.nlm.nih.gov/sites/entrez?Db=gene&Cmd=ShowDetailView&TermToSearch=1641) | doublecortex; lissencephaly, X-linked (doublecortin) |
| [SHANK3](http://www.ncbi.nlm.nih.gov/sites/entrez?Db=gene&Cmd=ShowDetailView&TermToSearch=85358) | SH3 and multiple ankyrin repeat domains 3 |
| [PTPRT](http://www.ncbi.nlm.nih.gov/sites/entrez?Db=gene&Cmd=ShowDetailView&TermToSearch=11122) | protein tyrosine phosphatase, receptor type, T |
| [TMEM35](http://www.ncbi.nlm.nih.gov/sites/entrez?Db=gene&Cmd=ShowDetailView&TermToSearch=59353) | transmembrane protein 35 |
| [FAM98C](http://www.ncbi.nlm.nih.gov/sites/entrez?Db=gene&Cmd=ShowDetailView&TermToSearch=147965) | family with sequence similarity 98, member C |
| [ISG20L2](http://www.ncbi.nlm.nih.gov/sites/entrez?Db=gene&Cmd=ShowDetailView&TermToSearch=81875) | interferon stimulated exonuclease gene 20kDa-like 2 |
| [RNF115](http://www.ncbi.nlm.nih.gov/sites/entrez?Db=gene&Cmd=ShowDetailView&TermToSearch=27246) | ring finger protein 115 |
| [NHLRC1](http://www.ncbi.nlm.nih.gov/sites/entrez?Db=gene&Cmd=ShowDetailView&TermToSearch=378884) | NHL repeat containing 1 |
| [FAM133B](http://www.ncbi.nlm.nih.gov/sites/entrez?Db=gene&Cmd=ShowDetailView&TermToSearch=257415) | family with sequence similarity 133, member B |
| [C18orf34](http://www.ncbi.nlm.nih.gov/sites/entrez?Db=gene&Cmd=ShowDetailView&TermToSearch=374864) | chromosome 18 open reading frame 34 |
| [CA6](http://www.ncbi.nlm.nih.gov/sites/entrez?Db=gene&Cmd=ShowDetailView&TermToSearch=765) | carbonic anhydrase VI |
| [DMD](http://www.ncbi.nlm.nih.gov/sites/entrez?Db=gene&Cmd=ShowDetailView&TermToSearch=1756) | dystrophin (muscular dystrophy, Duchenne and Becker types) |
| [MFHAS1](http://www.ncbi.nlm.nih.gov/sites/entrez?Db=gene&Cmd=ShowDetailView&TermToSearch=9258) | malignant fibrous histiocytoma amplified sequence 1 |
| [ZNF646](http://www.ncbi.nlm.nih.gov/sites/entrez?Db=gene&Cmd=ShowDetailView&TermToSearch=9726) | zinc finger protein 646 |
| [USP21](http://www.ncbi.nlm.nih.gov/sites/entrez?Db=gene&Cmd=ShowDetailView&TermToSearch=27005) | ubiquitin specific peptidase 21 |
| [VTI1A](http://www.ncbi.nlm.nih.gov/sites/entrez?Db=gene&Cmd=ShowDetailView&TermToSearch=143187) | vesicle transport through interaction with t-SNAREs homolog 1A (yeast) |
| [C18orf1](http://www.ncbi.nlm.nih.gov/sites/entrez?Db=gene&Cmd=ShowDetailView&TermToSearch=753) | chromosome 18 open reading frame 1 |
| [PFN2](http://www.ncbi.nlm.nih.gov/sites/entrez?Db=gene&Cmd=ShowDetailView&TermToSearch=5217) | profilin 2 |
| [KHDRBS2](http://www.ncbi.nlm.nih.gov/sites/entrez?Db=gene&Cmd=ShowDetailView&TermToSearch=202559) | KH domain containing, RNA binding, signal transduction associated 2 |
| [HIF1AN](http://www.ncbi.nlm.nih.gov/sites/entrez?Db=gene&Cmd=ShowDetailView&TermToSearch=55662) | hypoxia-inducible factor 1, alpha subunit inhibitor |
| [ERLIN1](http://www.ncbi.nlm.nih.gov/sites/entrez?Db=gene&Cmd=ShowDetailView&TermToSearch=10613) | ER lipid raft associated 1 |
| [LRP4](http://www.ncbi.nlm.nih.gov/sites/entrez?Db=gene&Cmd=ShowDetailView&TermToSearch=4038) | low density lipoprotein receptor-related protein 4 |
| [SRC](http://www.ncbi.nlm.nih.gov/sites/entrez?Db=gene&Cmd=ShowDetailView&TermToSearch=6714) | v-src sarcoma (Schmidt-Ruppin A-2) viral oncogene homolog (avian) |
| [USP6](http://www.ncbi.nlm.nih.gov/sites/entrez?Db=gene&Cmd=ShowDetailView&TermToSearch=9098) | ubiquitin specific peptidase 6 (Tre-2 oncogene) |
| [CPEB2](http://www.ncbi.nlm.nih.gov/sites/entrez?Db=gene&Cmd=ShowDetailView&TermToSearch=132864) | cytoplasmic polyadenylation element binding protein 2 |
| [SCN5A](http://www.ncbi.nlm.nih.gov/sites/entrez?Db=gene&Cmd=ShowDetailView&TermToSearch=6331) | sodium channel, voltage-gated, type V, alpha subunit |
| [KIAA1267](http://www.ncbi.nlm.nih.gov/sites/entrez?Db=gene&Cmd=ShowDetailView&TermToSearch=284058) | KIAA1267 |
| [FBXW11](http://www.ncbi.nlm.nih.gov/sites/entrez?Db=gene&Cmd=ShowDetailView&TermToSearch=23291) | F-box and WD repeat domain containing 11 |
| [DIP2B](http://www.ncbi.nlm.nih.gov/sites/entrez?Db=gene&Cmd=ShowDetailView&TermToSearch=57609) | DIP2 disco-interacting protein 2 homolog B (Drosophila) |
| [GATA4](http://www.ncbi.nlm.nih.gov/sites/entrez?Db=gene&Cmd=ShowDetailView&TermToSearch=2626) | GATA binding protein 4 |
| [GPT2](http://www.ncbi.nlm.nih.gov/sites/entrez?Db=gene&Cmd=ShowDetailView&TermToSearch=84706) | glutamic pyruvate transaminase (alanine aminotransferase) 2 |
| [GRIA4](http://www.ncbi.nlm.nih.gov/sites/entrez?Db=gene&Cmd=ShowDetailView&TermToSearch=2893) | glutamate receptor, ionotrophic, AMPA 4 |
| [UBAP2L](http://www.ncbi.nlm.nih.gov/sites/entrez?Db=gene&Cmd=ShowDetailView&TermToSearch=9898) | ubiquitin associated protein 2-like |
| [PARD6B](http://www.ncbi.nlm.nih.gov/sites/entrez?Db=gene&Cmd=ShowDetailView&TermToSearch=84612) | par-6 partitioning defective 6 homolog beta (C. elegans) |
| [MEX3A](http://www.ncbi.nlm.nih.gov/sites/entrez?Db=gene&Cmd=ShowDetailView&TermToSearch=92312) | mex-3 homolog A (C. elegans) |
| [PIGX](http://www.ncbi.nlm.nih.gov/sites/entrez?Db=gene&Cmd=ShowDetailView&TermToSearch=54965) | phosphatidylinositol glycan anchor biosynthesis, class X |
| [CHST1](http://www.ncbi.nlm.nih.gov/sites/entrez?Db=gene&Cmd=ShowDetailView&TermToSearch=8534) | carbohydrate (keratan sulfate Gal-6) sulfotransferase 1 |
| [H3F3B](http://www.ncbi.nlm.nih.gov/sites/entrez?Db=gene&Cmd=ShowDetailView&TermToSearch=3021) | H3 histone, family 3B (H3.3B) |
| [LAMC1](http://www.ncbi.nlm.nih.gov/sites/entrez?Db=gene&Cmd=ShowDetailView&TermToSearch=3915) | laminin, gamma 1 (formerly LAMB2) |
| [AGPAT5](http://www.ncbi.nlm.nih.gov/sites/entrez?Db=gene&Cmd=ShowDetailView&TermToSearch=55326) | 1-acylglycerol-3-phosphate O-acyltransferase 5 (lysophosphatidic acid acyltransferase, epsilon) |
| [MAPK1IP1L](http://www.ncbi.nlm.nih.gov/sites/entrez?Db=gene&Cmd=ShowDetailView&TermToSearch=93487) | mitogen-activated protein kinase 1 interacting protein 1-like |
| [MIER1](http://www.ncbi.nlm.nih.gov/sites/entrez?Db=gene&Cmd=ShowDetailView&TermToSearch=57708) | mesoderm induction early response 1 homolog (Xenopus laevis) |
| [RASGRP4](http://www.ncbi.nlm.nih.gov/sites/entrez?Db=gene&Cmd=ShowDetailView&TermToSearch=115727) | RAS guanyl releasing protein 4 |
| [FOXN3](http://www.ncbi.nlm.nih.gov/sites/entrez?Db=gene&Cmd=ShowDetailView&TermToSearch=1112) | forkhead box N3 |
| [B4GALT5](http://www.ncbi.nlm.nih.gov/sites/entrez?Db=gene&Cmd=ShowDetailView&TermToSearch=9334) | UDP-Gal:betaGlcNAc beta 1,4- galactosyltransferase, polypeptide 5 |
| [PRPF40A](http://www.ncbi.nlm.nih.gov/sites/entrez?Db=gene&Cmd=ShowDetailView&TermToSearch=55660) | PRP40 pre-mRNA processing factor 40 homolog A (S. cerevisiae) |
| [DMRT1](http://www.ncbi.nlm.nih.gov/sites/entrez?Db=gene&Cmd=ShowDetailView&TermToSearch=1761) | doublesex and mab-3 related transcription factor 1 |
| [CACNB2](http://www.ncbi.nlm.nih.gov/sites/entrez?Db=gene&Cmd=ShowDetailView&TermToSearch=783) | calcium channel, voltage-dependent, beta 2 subunit |
| [FAM178A](http://www.ncbi.nlm.nih.gov/sites/entrez?Db=gene&Cmd=ShowDetailView&TermToSearch=55719) | family with sequence similarity 178, member A |
| [SP2](http://www.ncbi.nlm.nih.gov/sites/entrez?Db=gene&Cmd=ShowDetailView&TermToSearch=6668) | Sp2 transcription factor |
| [VPS35](http://www.ncbi.nlm.nih.gov/sites/entrez?Db=gene&Cmd=ShowDetailView&TermToSearch=55737) | vacuolar protein sorting 35 homolog (S. cerevisiae) |
| [SHOX2](http://www.ncbi.nlm.nih.gov/sites/entrez?Db=gene&Cmd=ShowDetailView&TermToSearch=6474) | short stature homeobox 2 |
| [FLJ21865](http://www.ncbi.nlm.nih.gov/sites/entrez?Db=gene&Cmd=ShowDetailView&TermToSearch=64772) | endo-beta-N-acetylglucosaminidase |
| [ARID2](http://www.ncbi.nlm.nih.gov/sites/entrez?Db=gene&Cmd=ShowDetailView&TermToSearch=196528) | AT rich interactive domain 2 (ARID, RFX-like) |
| [ZFP91](http://www.ncbi.nlm.nih.gov/sites/entrez?Db=gene&Cmd=ShowDetailView&TermToSearch=80829) | zinc finger protein 91 homolog (mouse) |
| [STAG3L4](http://www.ncbi.nlm.nih.gov/sites/entrez?Db=gene&Cmd=ShowDetailView&TermToSearch=64940) | stromal antigen 3-like 4 |
| [CTNND2](http://www.ncbi.nlm.nih.gov/sites/entrez?Db=gene&Cmd=ShowDetailView&TermToSearch=1501) | catenin (cadherin-associated protein), delta 2 (neural plakophilin-related arm-repeat protein) |
| [NCAM1](http://www.ncbi.nlm.nih.gov/sites/entrez?Db=gene&Cmd=ShowDetailView&TermToSearch=4684) | neural cell adhesion molecule 1 |
| [GK5](http://www.ncbi.nlm.nih.gov/sites/entrez?Db=gene&Cmd=ShowDetailView&TermToSearch=256356) | glycerol kinase 5 (putative) |
| [FOXN4](http://www.ncbi.nlm.nih.gov/sites/entrez?Db=gene&Cmd=ShowDetailView&TermToSearch=121643) | forkhead box N4 |
| [TIMP3](http://www.ncbi.nlm.nih.gov/sites/entrez?Db=gene&Cmd=ShowDetailView&TermToSearch=7078) | TIMP metallopeptidase inhibitor 3 (Sorsby fundus dystrophy, pseudoinflammatory) |
| [CENPP](http://www.ncbi.nlm.nih.gov/sites/entrez?Db=gene&Cmd=ShowDetailView&TermToSearch=401541) | centromere protein P |
| [SPN](http://www.ncbi.nlm.nih.gov/sites/entrez?Db=gene&Cmd=ShowDetailView&TermToSearch=6693) | sialophorin (leukosialin, CD43) |
| [TARSL2](http://www.ncbi.nlm.nih.gov/sites/entrez?Db=gene&Cmd=ShowDetailView&TermToSearch=123283) | threonyl-tRNA synthetase-like 2 |
| [CNOT1](http://www.ncbi.nlm.nih.gov/sites/entrez?Db=gene&Cmd=ShowDetailView&TermToSearch=23019) | CCR4-NOT transcription complex, subunit 1 |
| [ZIC1](http://www.ncbi.nlm.nih.gov/sites/entrez?Db=gene&Cmd=ShowDetailView&TermToSearch=7545) | Zic family member 1 (odd-paired homolog, Drosophila) |
| [PAPOLB](http://www.ncbi.nlm.nih.gov/sites/entrez?Db=gene&Cmd=ShowDetailView&TermToSearch=56903) | poly(A) polymerase beta (testis specific) |
| [IPPK](http://www.ncbi.nlm.nih.gov/sites/entrez?Db=gene&Cmd=ShowDetailView&TermToSearch=64768) | inositol 1,3,4,5,6-pentakisphosphate 2-kinase |
| [C10orf54](http://www.ncbi.nlm.nih.gov/sites/entrez?Db=gene&Cmd=ShowDetailView&TermToSearch=64115) | chromosome 10 open reading frame 54 |
| [MAPK1](http://www.ncbi.nlm.nih.gov/sites/entrez?Db=gene&Cmd=ShowDetailView&TermToSearch=5594) | mitogen-activated protein kinase 1 |
| [CLLU1](http://www.ncbi.nlm.nih.gov/sites/entrez?Db=gene&Cmd=ShowDetailView&TermToSearch=574028) | chronic lymphocytic leukemia up-regulated 1 |
| [ST8SIA4](http://www.ncbi.nlm.nih.gov/sites/entrez?Db=gene&Cmd=ShowDetailView&TermToSearch=7903) | ST8 alpha-N-acetyl-neuraminide alpha-2,8-sialyltransferase 4 |
| [CCDC76](http://www.ncbi.nlm.nih.gov/sites/entrez?Db=gene&Cmd=ShowDetailView&TermToSearch=54482) | coiled-coil domain containing 76 |

| **miR-571** |  |
| --- | --- |
| **Target Gene** | **Gene name** |
| [STK36](http://www.ncbi.nlm.nih.gov/sites/entrez?Db=gene&Cmd=ShowDetailView&TermToSearch=27148) | serine/threonine kinase 36, fused homolog (Drosophila) |
| [GRID1](http://www.ncbi.nlm.nih.gov/sites/entrez?Db=gene&Cmd=ShowDetailView&TermToSearch=2894) | glutamate receptor, ionotropic, delta 1 |
| [ZWINT](http://www.ncbi.nlm.nih.gov/sites/entrez?Db=gene&Cmd=ShowDetailView&TermToSearch=11130) | ZW10 interactor |
| [SGTB](http://www.ncbi.nlm.nih.gov/sites/entrez?Db=gene&Cmd=ShowDetailView&TermToSearch=54557) | small glutamine-rich tetratricopeptide repeat (TPR)-containing, beta |
| [LAMA1](http://www.ncbi.nlm.nih.gov/sites/entrez?Db=gene&Cmd=ShowDetailView&TermToSearch=284217) | laminin, alpha 1 |
| [SPAG8](http://www.ncbi.nlm.nih.gov/sites/entrez?Db=gene&Cmd=ShowDetailView&TermToSearch=26206) | sperm associated antigen 8 |
| [CREBBP](http://www.ncbi.nlm.nih.gov/sites/entrez?Db=gene&Cmd=ShowDetailView&TermToSearch=1387) | CREB binding protein (Rubinstein-Taybi syndrome) |
| [CYP24A1](http://www.ncbi.nlm.nih.gov/sites/entrez?Db=gene&Cmd=ShowDetailView&TermToSearch=1591) | cytochrome P450, family 24, subfamily A, polypeptide 1 |
| [SCRT2](http://www.ncbi.nlm.nih.gov/sites/entrez?Db=gene&Cmd=ShowDetailView&TermToSearch=85508) | scratch homolog 2, zinc finger protein (Drosophila) |
| [RANBP10](http://www.ncbi.nlm.nih.gov/sites/entrez?Db=gene&Cmd=ShowDetailView&TermToSearch=57610) | RAN binding protein 10 |
| [FLJ20160](http://www.ncbi.nlm.nih.gov/sites/entrez?Db=gene&Cmd=ShowDetailView&TermToSearch=54842) | FLJ20160 protein |
| [OTUD3](http://www.ncbi.nlm.nih.gov/sites/entrez?Db=gene&Cmd=ShowDetailView&TermToSearch=23252) | OTU domain containing 3 |
| [PAPOLA](http://www.ncbi.nlm.nih.gov/sites/entrez?Db=gene&Cmd=ShowDetailView&TermToSearch=10914) | poly(A) polymerase alpha |
| [HIP1](http://www.ncbi.nlm.nih.gov/sites/entrez?Db=gene&Cmd=ShowDetailView&TermToSearch=3092) | huntingtin interacting protein 1 |
| [KIAA0232](http://www.ncbi.nlm.nih.gov/sites/entrez?Db=gene&Cmd=ShowDetailView&TermToSearch=9778) | KIAA0232 |
| [SPATA2](http://www.ncbi.nlm.nih.gov/sites/entrez?Db=gene&Cmd=ShowDetailView&TermToSearch=9825) | spermatogenesis associated 2 |
| [CTDSPL](http://www.ncbi.nlm.nih.gov/sites/entrez?Db=gene&Cmd=ShowDetailView&TermToSearch=10217) | CTD (carboxy-terminal domain, RNA polymerase II, polypeptide A) small phosphatase-like |
| [DSCAML1](http://www.ncbi.nlm.nih.gov/sites/entrez?Db=gene&Cmd=ShowDetailView&TermToSearch=57453) | Down syndrome cell adhesion molecule like 1 |
| [CHL1](http://www.ncbi.nlm.nih.gov/sites/entrez?Db=gene&Cmd=ShowDetailView&TermToSearch=10752) | cell adhesion molecule with homology to L1CAM (close homolog of L1) |
| [CHD1](http://www.ncbi.nlm.nih.gov/sites/entrez?Db=gene&Cmd=ShowDetailView&TermToSearch=1105) | chromodomain helicase DNA binding protein 1 |
| [KCNC1](http://www.ncbi.nlm.nih.gov/sites/entrez?Db=gene&Cmd=ShowDetailView&TermToSearch=3746) | potassium voltage-gated channel, Shaw-related subfamily, member 1 |
| [LINGO1](http://www.ncbi.nlm.nih.gov/sites/entrez?Db=gene&Cmd=ShowDetailView&TermToSearch=84894) | leucine rich repeat and Ig domain containing 1 |
| [ZFP64](http://www.ncbi.nlm.nih.gov/sites/entrez?Db=gene&Cmd=ShowDetailView&TermToSearch=55734) | zinc finger protein 64 homolog (mouse) |
| [GTDC1](http://www.ncbi.nlm.nih.gov/sites/entrez?Db=gene&Cmd=ShowDetailView&TermToSearch=79712) | glycosyltransferase-like domain containing 1 |
| [RAP1A](http://www.ncbi.nlm.nih.gov/sites/entrez?Db=gene&Cmd=ShowDetailView&TermToSearch=5906) | RAP1A, member of RAS oncogene family |
| [DNAL1](http://www.ncbi.nlm.nih.gov/sites/entrez?Db=gene&Cmd=ShowDetailView&TermToSearch=83544) | dynein, axonemal, light chain 1 |
| [ST8SIA4](http://www.ncbi.nlm.nih.gov/sites/entrez?Db=gene&Cmd=ShowDetailView&TermToSearch=7903) | ST8 alpha-N-acetyl-neuraminide alpha-2,8-sialyltransferase 4 |
| [FKBP5](http://www.ncbi.nlm.nih.gov/sites/entrez?Db=gene&Cmd=ShowDetailView&TermToSearch=2289) | FK506 binding protein 5 |
| [CAMSAP1L1](http://www.ncbi.nlm.nih.gov/sites/entrez?Db=gene&Cmd=ShowDetailView&TermToSearch=23271) | calmodulin regulated spectrin-associated protein 1-like 1 |
| [XPO1](http://www.ncbi.nlm.nih.gov/sites/entrez?Db=gene&Cmd=ShowDetailView&TermToSearch=7514) | exportin 1 (CRM1 homolog, yeast) |
| [SPSB1](http://www.ncbi.nlm.nih.gov/sites/entrez?Db=gene&Cmd=ShowDetailView&TermToSearch=80176) | splA/ryanodine receptor domain and SOCS box containing 1 |
| [DTX4](http://www.ncbi.nlm.nih.gov/sites/entrez?Db=gene&Cmd=ShowDetailView&TermToSearch=23220) | deltex 4 homolog (Drosophila) |
| [ABLIM3](http://www.ncbi.nlm.nih.gov/sites/entrez?Db=gene&Cmd=ShowDetailView&TermToSearch=22885) | actin binding LIM protein family, member 3 |
| [ONECUT2](http://www.ncbi.nlm.nih.gov/sites/entrez?Db=gene&Cmd=ShowDetailView&TermToSearch=9480) | one cut homeobox 2 |
| [GARNL4](http://www.ncbi.nlm.nih.gov/sites/entrez?Db=gene&Cmd=ShowDetailView&TermToSearch=23108) | GTPase activating Rap/RanGAP domain-like 4 |
| [SYVN1](http://www.ncbi.nlm.nih.gov/sites/entrez?Db=gene&Cmd=ShowDetailView&TermToSearch=84447) | synovial apoptosis inhibitor 1, synoviolin |
| [TNRC6B](http://www.ncbi.nlm.nih.gov/sites/entrez?Db=gene&Cmd=ShowDetailView&TermToSearch=23112) | trinucleotide repeat containing 6B |
| [FOXP2](http://www.ncbi.nlm.nih.gov/sites/entrez?Db=gene&Cmd=ShowDetailView&TermToSearch=93986) | forkhead box P2 |
| [ELAVL1](http://www.ncbi.nlm.nih.gov/sites/entrez?Db=gene&Cmd=ShowDetailView&TermToSearch=1994) | ELAV (embryonic lethal, abnormal vision, Drosophila)-like 1 (Hu antigen R) |
| [VTI1A](http://www.ncbi.nlm.nih.gov/sites/entrez?Db=gene&Cmd=ShowDetailView&TermToSearch=143187) | vesicle transport through interaction with t-SNAREs homolog 1A (yeast) |
| [ZNF607](http://www.ncbi.nlm.nih.gov/sites/entrez?Db=gene&Cmd=ShowDetailView&TermToSearch=84775) | zinc finger protein 607 |
| [AFF1](http://www.ncbi.nlm.nih.gov/sites/entrez?Db=gene&Cmd=ShowDetailView&TermToSearch=4299) | AF4/FMR2 family, member 1 |
| [NAV1](http://www.ncbi.nlm.nih.gov/sites/entrez?Db=gene&Cmd=ShowDetailView&TermToSearch=89796) | neuron navigator 1 |
| [ZFHX4](http://www.ncbi.nlm.nih.gov/sites/entrez?Db=gene&Cmd=ShowDetailView&TermToSearch=79776) | zinc finger homeobox 4 |
| [POU3F1](http://www.ncbi.nlm.nih.gov/sites/entrez?Db=gene&Cmd=ShowDetailView&TermToSearch=5453) | POU class 3 homeobox 1 |
| [SLC6A20](http://www.ncbi.nlm.nih.gov/sites/entrez?Db=gene&Cmd=ShowDetailView&TermToSearch=54716) | solute carrier family 6 (proline IMINO transporter), member 20 |
| [MLL2](http://www.ncbi.nlm.nih.gov/sites/entrez?Db=gene&Cmd=ShowDetailView&TermToSearch=8085) | myeloid/lymphoid or mixed-lineage leukemia 2 |
| [ANKRD42](http://www.ncbi.nlm.nih.gov/sites/entrez?Db=gene&Cmd=ShowDetailView&TermToSearch=338699) | ankyrin repeat domain 42 |
| [STX17](http://www.ncbi.nlm.nih.gov/sites/entrez?Db=gene&Cmd=ShowDetailView&TermToSearch=55014) | syntaxin 17 |
| [EIF5A2](http://www.ncbi.nlm.nih.gov/sites/entrez?Db=gene&Cmd=ShowDetailView&TermToSearch=56648) | eukaryotic translation initiation factor 5A2 |
| [FRAS1](http://www.ncbi.nlm.nih.gov/sites/entrez?Db=gene&Cmd=ShowDetailView&TermToSearch=80144) | Fraser syndrome 1 |
| [KIAA1324L](http://www.ncbi.nlm.nih.gov/sites/entrez?Db=gene&Cmd=ShowDetailView&TermToSearch=222223) | KIAA1324-like |
| [SH3BP5L](http://www.ncbi.nlm.nih.gov/sites/entrez?Db=gene&Cmd=ShowDetailView&TermToSearch=80851) | SH3-binding domain protein 5-like |
| [SOX4](http://www.ncbi.nlm.nih.gov/sites/entrez?Db=gene&Cmd=ShowDetailView&TermToSearch=6659) | SRY (sex determining region Y)-box 4 |
| [CTF8](http://www.ncbi.nlm.nih.gov/sites/entrez?Db=gene&Cmd=ShowDetailView&TermToSearch=54921) | chromosome transmission fidelity factor 8 homolog (S. cerevisiae) |
| [HMGB1](http://www.ncbi.nlm.nih.gov/sites/entrez?Db=gene&Cmd=ShowDetailView&TermToSearch=3146) | high-mobility group box 1 |
| [SLC4A4](http://www.ncbi.nlm.nih.gov/sites/entrez?Db=gene&Cmd=ShowDetailView&TermToSearch=8671) | solute carrier family 4, sodium bicarbonate cotransporter, member 4 |
| [C10orf46](http://www.ncbi.nlm.nih.gov/sites/entrez?Db=gene&Cmd=ShowDetailView&TermToSearch=143384) | chromosome 10 open reading frame 46 |
| [WIPF2](http://www.ncbi.nlm.nih.gov/sites/entrez?Db=gene&Cmd=ShowDetailView&TermToSearch=147179) | WAS/WASL interacting protein family, member 2 |
| [FBXW7](http://www.ncbi.nlm.nih.gov/sites/entrez?Db=gene&Cmd=ShowDetailView&TermToSearch=55294) | F-box and WD repeat domain containing 7 |
| [HMGCL](http://www.ncbi.nlm.nih.gov/sites/entrez?Db=gene&Cmd=ShowDetailView&TermToSearch=3155) | 3-hydroxymethyl-3-methylglutaryl-Coenzyme A lyase (hydroxymethylglutaricaciduria) |
| [TCF4](http://www.ncbi.nlm.nih.gov/sites/entrez?Db=gene&Cmd=ShowDetailView&TermToSearch=6925) | transcription factor 4 |
| [FIGNL2](http://www.ncbi.nlm.nih.gov/sites/entrez?Db=gene&Cmd=ShowDetailView&TermToSearch=401720) | fidgetin-like 2 |
| [PDXDC1](http://www.ncbi.nlm.nih.gov/sites/entrez?Db=gene&Cmd=ShowDetailView&TermToSearch=23042) | pyridoxal-dependent decarboxylase domain containing 1 |
| [NR2C2](http://www.ncbi.nlm.nih.gov/sites/entrez?Db=gene&Cmd=ShowDetailView&TermToSearch=7182) | nuclear receptor subfamily 2, group C, member 2 |
| [ZSWIM4](http://www.ncbi.nlm.nih.gov/sites/entrez?Db=gene&Cmd=ShowDetailView&TermToSearch=65249) | zinc finger, SWIM-type containing 4 |
| [NUFIP2](http://www.ncbi.nlm.nih.gov/sites/entrez?Db=gene&Cmd=ShowDetailView&TermToSearch=57532) | nuclear fragile X mental retardation protein interacting protein 2 |
| [DTX1](http://www.ncbi.nlm.nih.gov/sites/entrez?Db=gene&Cmd=ShowDetailView&TermToSearch=1840) | deltex homolog 1 (Drosophila) |
| [GK](http://www.ncbi.nlm.nih.gov/sites/entrez?Db=gene&Cmd=ShowDetailView&TermToSearch=2710) | glycerol kinase |
| [YBX2](http://www.ncbi.nlm.nih.gov/sites/entrez?Db=gene&Cmd=ShowDetailView&TermToSearch=51087) | Y box binding protein 2 |

| **miR-652** |  |
| --- | --- |
| **Target Gene** | **Gene name** |
| [ISL1](http://www.ncbi.nlm.nih.gov/sites/entrez?Db=gene&Cmd=ShowDetailView&TermToSearch=3670) | ISL LIM homeobox 1 |
| [ACVR2B](http://www.ncbi.nlm.nih.gov/sites/entrez?Db=gene&Cmd=ShowDetailView&TermToSearch=93) | activin A receptor, type IIB |
| [PSKH1](http://www.ncbi.nlm.nih.gov/sites/entrez?Db=gene&Cmd=ShowDetailView&TermToSearch=5681) | protein serine kinase H1 |

**Supporting Table S5: Identified target genes of miR-513-3p, miR-571 and miR-652.** The miRNA databases and target prediction tools miRBase (http://microrna.sanger.ac.uk), TargetScan (http://www.targetscan.org) and microRNA.org – Targets and Expression (http://www.microrna.org) were used to identify the listed target genes.

**Supporting Materials and Methods**

**Quantitative real-time PCR.** For quantitative real-time PCR, the quantity and quality of the RNA was determined spectroscopically using a nanodrop (Thermo Scientific, Waltham, MA). cDNA was synthesized out of total RNA (1 µg) using miScript Reverse Transcriptase Kit (Qiagen) according to the manufacturer’s protocol, and was resuspended in suitable amounts of H_2_O. cDNA samples (2 µl) were used for real-time PCR in a total volume of 25 µl using the miScript SYBR Green PCR Kit (Qiagen) and miRNA specific primers (Qiagen) on a qPCR machine (Applied Biosystems 7300 Sequence Detection System, Applied Biosystems, Foster City, CA). All real-time PCR reactions were performed in duplicates. Unspecific reactions were excluded by using controls with double distilled water for each primer. Data were generated and analyzed using SDS 2.3 and RQ manager 1.2 software. All values were normalized to the U6 miRNA or SV40 for the serum samples as described in [1].

**miRNA-Isolation from tissue.** Total RNA was purified from liver tissue using Qiazol reagent (Invitrogen) and miRNeasy Mini kit (Qiagen) according to the manufacturer’s protocol, and was resuspended in suitable amounts of H_2_O.

**Cell Culture, Stimulation and Transfection.** The hepatocellular carcinoma cell line HuH-7 was cultured in Dulbecco’s modified Eagle’s medium with 10% fetal bovine serum, 4 mM L-glutamine and penicillin/streptomycin. The hepatic stellate cell line LX-2 was cultured in Dulbecco’s modified Eagle’s medium with only 2% fetal bovine serum, 5 mM L-glutamine and penicillin/streptomycin. For transfection the Hiperfect transfection reagent (Qiagen) with miRNA specific plasmids (Qiagen) was used according to the manufacturer’s instructions. Briefly, both of the two cell lines (HuH-7 and LX-2) were either transfected with indicated amounts of miR-571 mimetic (Qiagen) or just seeded in the unmodified medium as control. Forty-eight hours after transfection cells were harvested for RNA isolation, while proteins were isolated from cells that were transfected for ninety-six hours.

Lymphomic monocyte cell line U937 cells were cultured in RPMI medium with 10% fetal bovine serum, 300 mg/L L-glutamine and penicillin/streptomycin. For differentiation, cells were seeded on 6-well-plates and stimulated with 0,1 µg/ml PMA for 24 hours and further (1) left untreated; (2) stimulated for 48 h with 1 µg/ml LPS.

**I*n silico-*analysis of potential miRNA target genes.** The microRNA databases and target prediction tools miRBase (http://microrna.sanger.ac.uk/), TargetScan (http://www.targetscan.org/index.html) and mircrorna.org (http://www.microrna.org) were used to identify potential microRNA targets.

**Statistics.** Results of experimental data are expressed as the mean + standard error of the mean (SEM). Statistical significance between experimental groups was assessed using an unpaired two-sample Student’s t-test, and p values < 0.05 were considered as significant. Results of clinical data and miRNA serum concentrations from patients were expressed as median and range, due to skewed distributions of most variables in patients. Differences between controls and patients or between patient subgroups were analyzed by Mann-Whitney U-test, and correlations between serum levels of miRNA and clinical values were determined by calculating the spearman rank correlation coefficient.

Receiver operating characteristic (ROC) curve analysis and the derived c statistic provide a global and standardized appreciation of the accuracy of a marker or a composite score for predicting an event. This statistic allows a simple comparison of the accuracy of different prognostic scores within the same population. The ROC curve represents the plotting of sensitivity against 1-specificity. A c statistic of 0.5 means that discrimination is due to chance alone, a c statistic of 1 means that the score perfectly predicts outcome (a goal never achieved in clinical practice). Therefore, the accuracy of a score increases when c statistic moves from 0.5 to 1. Statistical analyzes regarding the clinical data were performed using SPSS software (version 12.0, SPSS Inc., Chicago, IL).

Differentially expressed miRNAs with significant P-values (0.05) were selected for cluster analysis using a hierarchical method with average linkage and Euclidean distance metric. Clustering plots were generated using TIGR MeV (Multiple Experimental Viewer) software from the Institute for Genomic Research.

**Supporting References**

1. Roderburg C, Urban GW, Bettermann K, Vucur M, Zimmermann H, Schmidt S, Janssen J, et al. Micro-RNA profiling reveals a role for miR-29 in human and murine liver fibrosis. Hepatology;53:209-218.
