# Supplementary figures and images for: Micro-RNA Profiling in Human Serum Reveals Compartment-Specific Roles of miR-571 and miR-652 in Liver Cirrhosis
Source: PLoS One. 2012 Mar 7;7(3):e32999. doi: 10.1371/journal.pone.0032999 (PMC3296762; doi:10.1371/journal.pone.0032999)

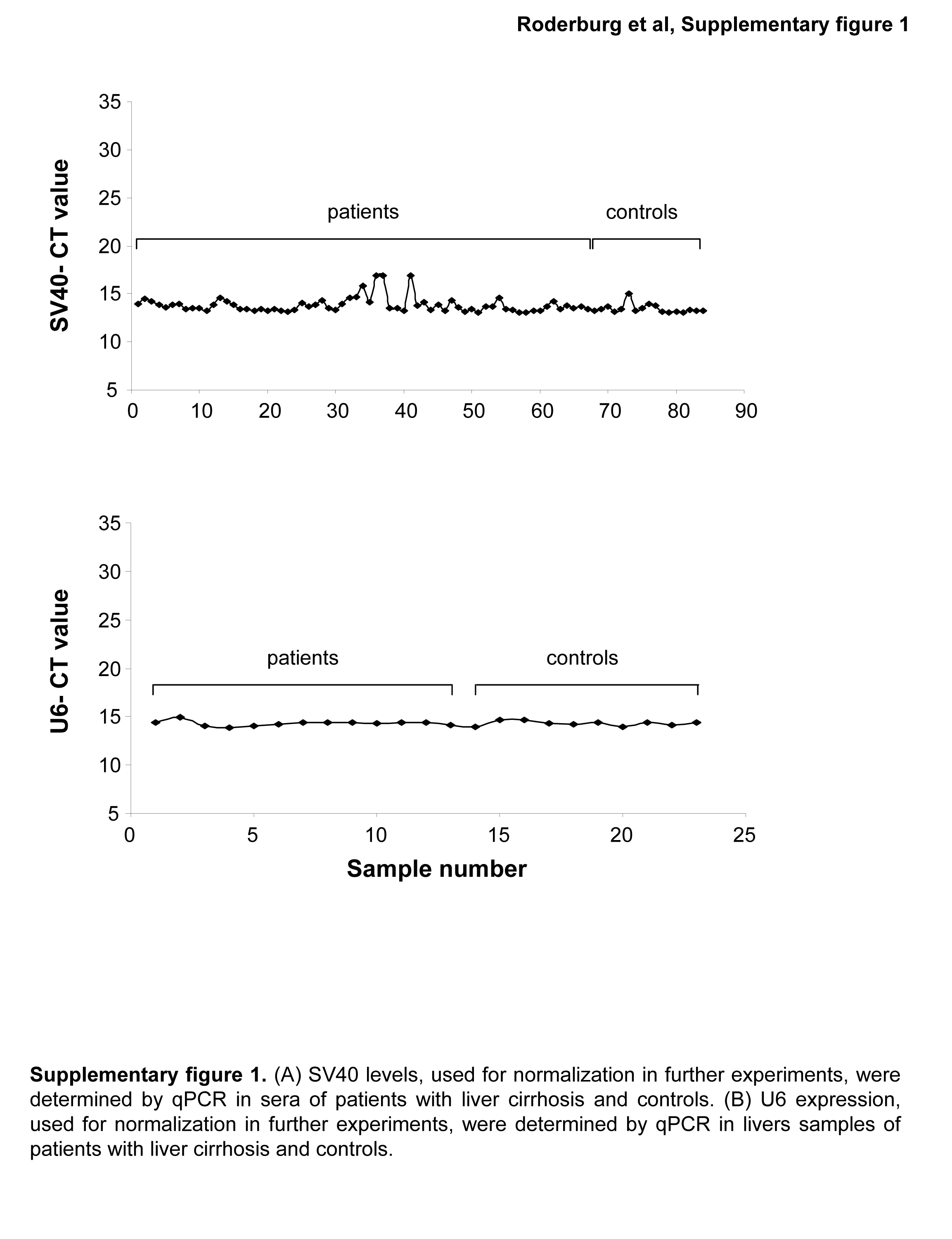

Supplement: Figure S1 — SV40 and U6 were used for normalization of PCR based analysis. (A) SV40 levels, used for normalization in further experiments, were determined by qPCR in sera of patients with liver cirrhosis and controls. (B) U6 expression, used for normalization in further experiments, were determined by qPCR in livers samples of patients with liver cirrhosis and controls. (TIF) [file pone.0032999.s002.tif]

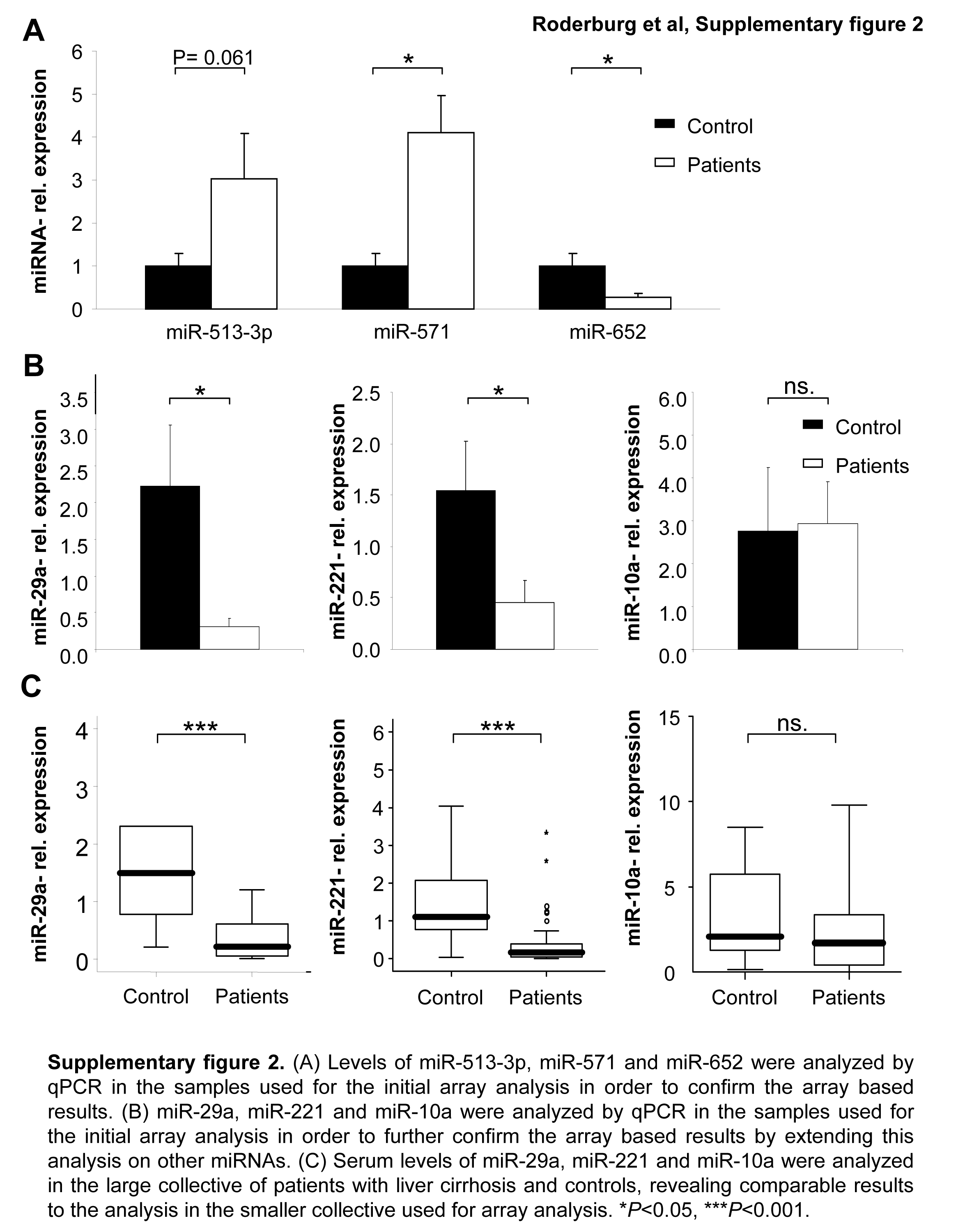

Supplement: Figure S2 — Confirmation of the array based results by using qPCR. (A) Levels of miR-513-3p, miR-571 and miR-652 were analyzed by qPCR in the samples used for the initial array analysis in order to confirm the array based results. (B) miR-29a, miR-221 and miR-10a were analyzed by qPCR in the samples used for the initial array analysis in order to further confirm the array based results by extending this analysis on other miRNAs. (C) Serum levels of miR-29a, miR-221 and miR-10a were analyzed in the large collective of patients with liver cirrhosis and controls, revealing comparable results to the analysis in the smaller collective used for array analysis. *P<0.05, ***P<0.001. (TIF) [file pone.0032999.s003.tif]

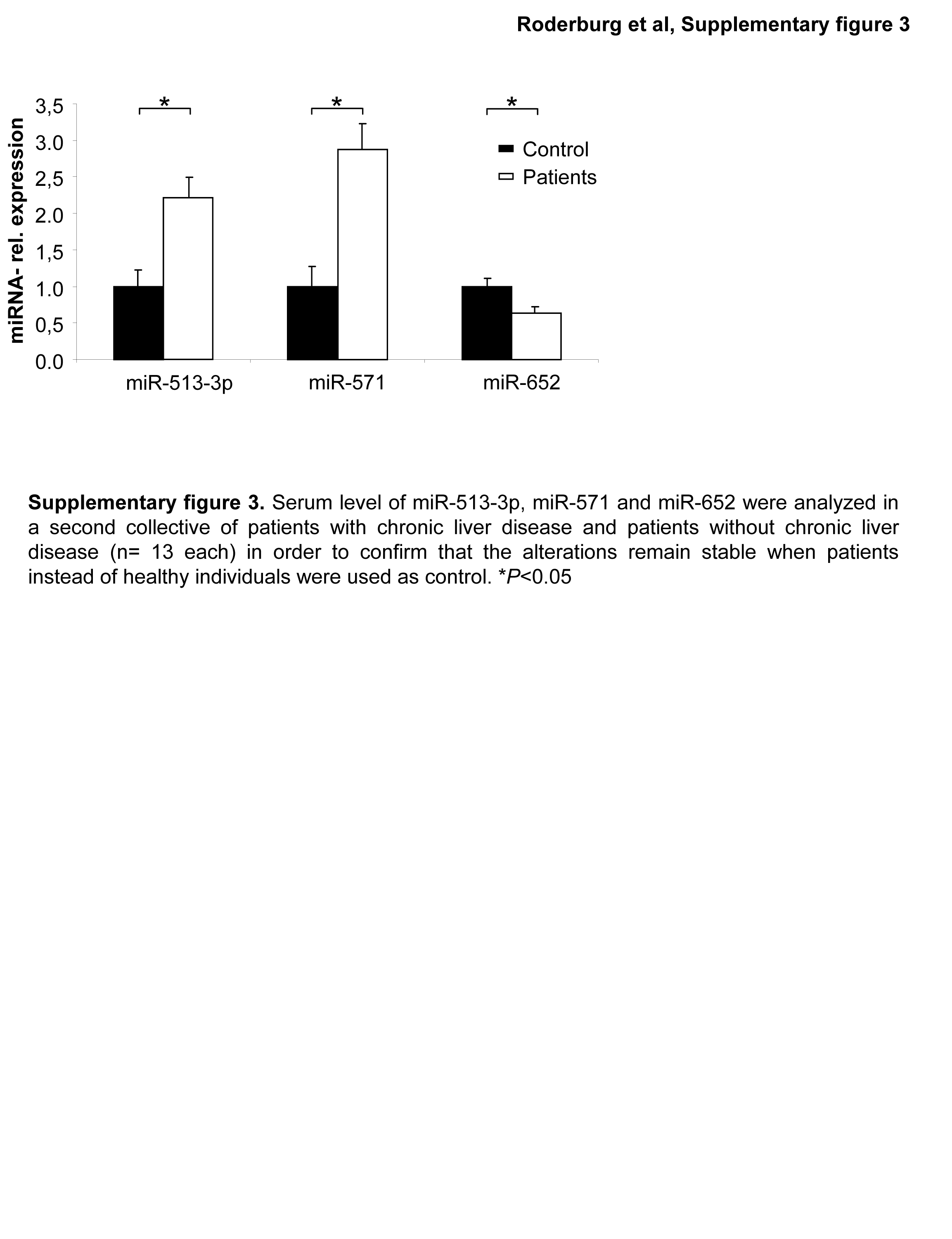

Supplement: Figure S3 — Confirmation of alterations of serum levels of miR-513-3p, miR-571 and miR-652 in a second cohort of patients. Serum level of miR-513-3p, miR-571 and miR-652 were analyzed in a second collective of patients with chronic liver disease and patients without chronic liver disease (n = 13 each) in order to confirm that the alterations remain stable when patients instead of healthy individuals were used as control. *P<0.05. (TIF) [file pone.0032999.s004.tif]

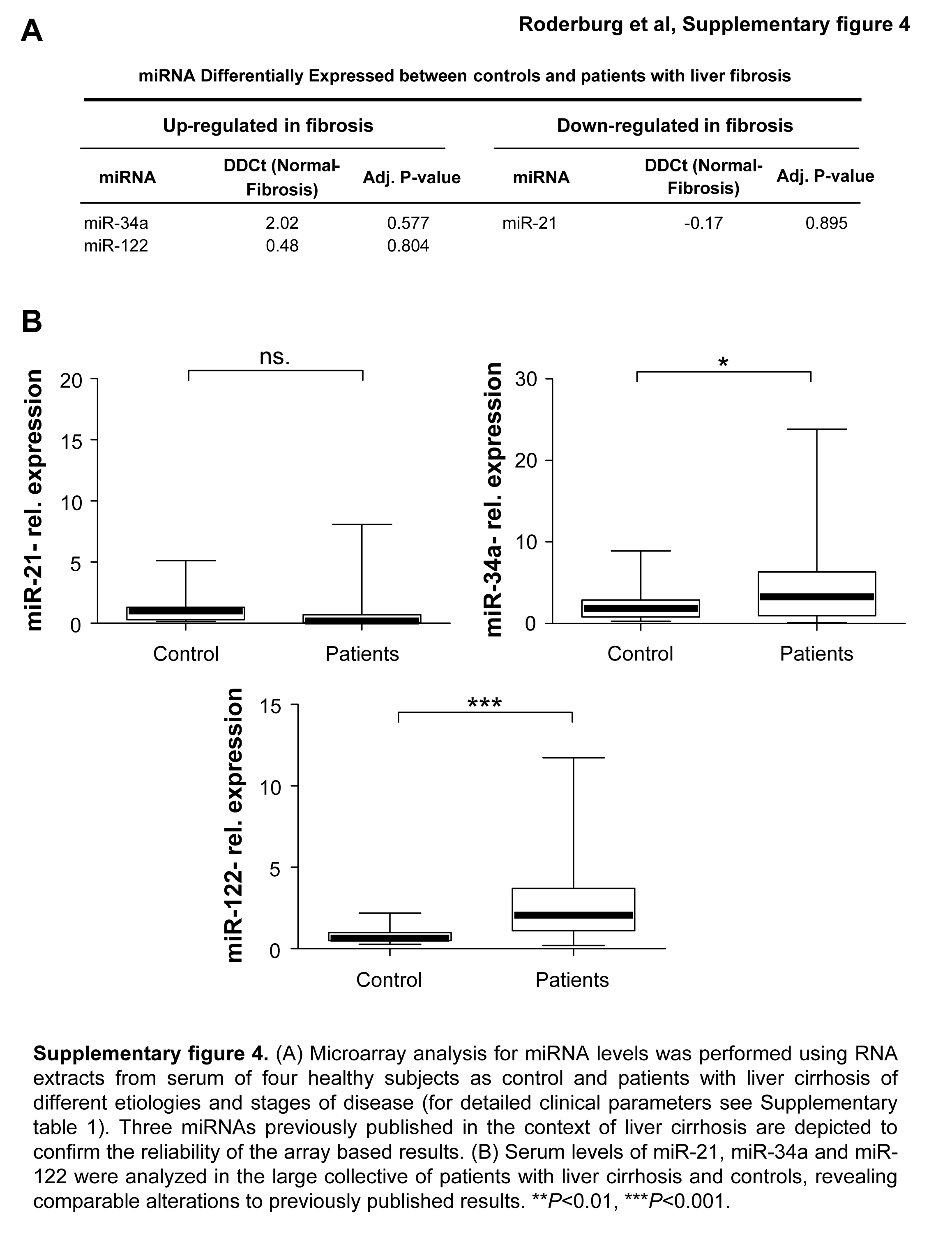

Supplement: Figure S4 — Validation of the array- and qPCR based data. (A) Microarray analysis for miRNA levels was performed using RNA extracts from serum of four healthy subjects as control and patients with liver cirrhosis of different etiologies and stages of disease (for detailed clinical parameters see Data S1). Three miRNAs previously published in the context of liver cirrhosis are depicted to confirm the reliability of the array based results. (B) Serum levels of miR-21, miR-34a and miR-122 were analyzed in the large collective of patients with liver cirrhosis and controls, revealing comparable alterations to previously published results. **P<0.01, ***P<0.001. (TIF) [file pone.0032999.s005.tif]

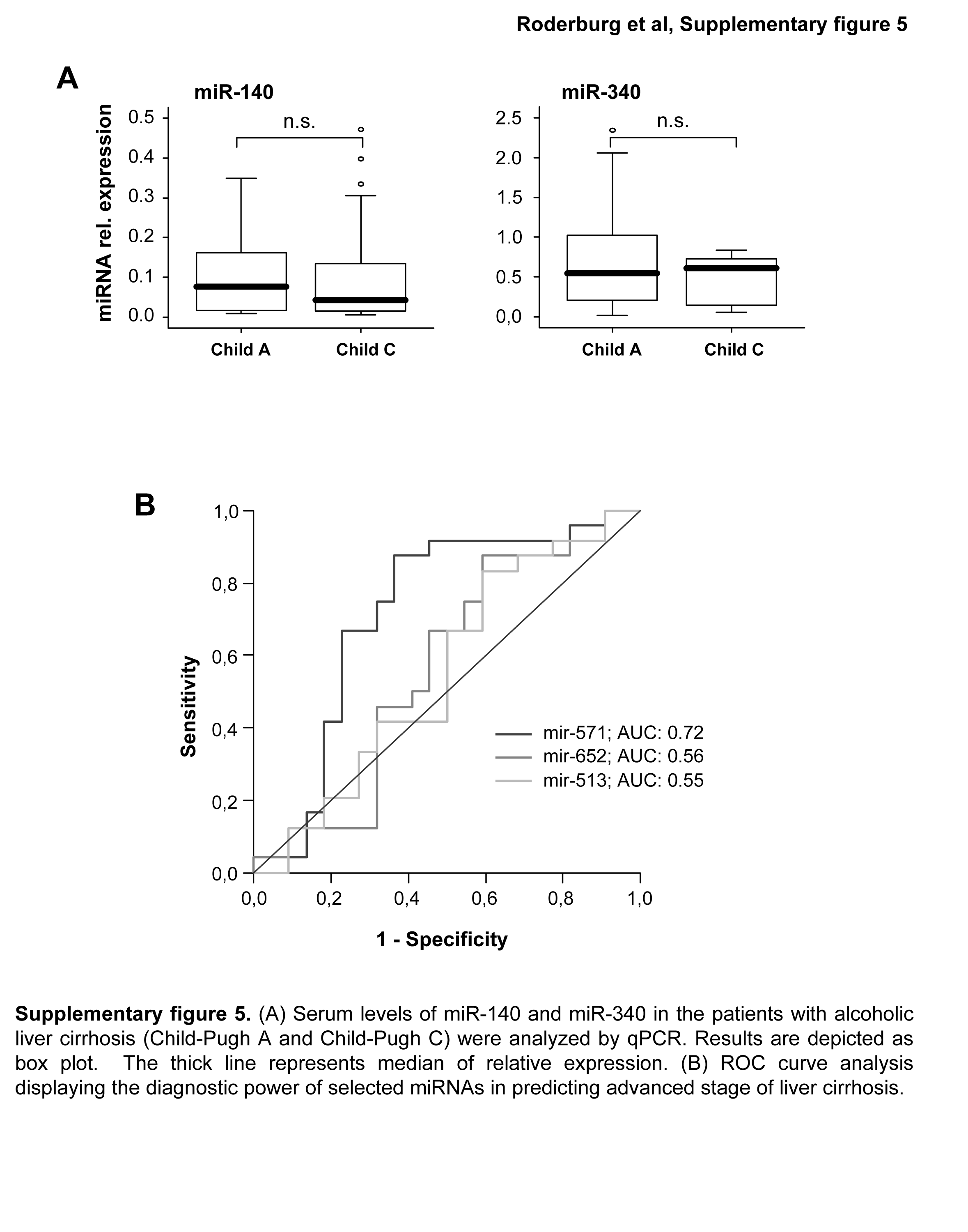

Supplement: Figure S5 — Analysis of miR-140 and miR-340 in the serum of patients with liver cirrhosis. (A) Serum levels of miR-140 and miR-340 in the patients with alcoholic liver cirrhosis (Child-Pugh A and Child-Pugh C) were analyzed by qPCR. Results are depicted as box plot. The thick line represents median of relative expression. (B) ROC curve analysis displaying the diagnostic power of selected miRNAs in predicting advanced stage of liver cirrhosis. (TIF) [file pone.0032999.s006.tif]
